# Synthesis of 5‐Alkyl‐ and 5‐Phenylamino‐Substituted Azothiazole Dyes with Solvatochromic and DNA‐Binding Properties

**DOI:** 10.1002/chem.201903657

**Published:** 2019-11-08

**Authors:** Phil M. Pithan, Christopher Kuhlmann, Carsten Engelhard, Heiko Ihmels

**Affiliations:** ^1^ Department of Chemistry and Biology, and Center of Micro- and Nanochemistry and Engineering University of Siegen Adolf-Reichwein-Str. 2 57068 Siegen Germany

**Keywords:** azothiazole dyes, DNA ligands, fluorescent probes, intercalation, solvatochromism

## Abstract

A series of new 5‐mono‐ and 5,5′‐bisamino‐substituted azothiazole derivatives was synthesized from the readily available diethyl azothiazole‐4,4′‐dicarboxylate. This reaction most likely comprises an initial Michael‐type addition by the respective primary alkyl and aromatic amines at the carbon atom C5 of the substrate. Subsequently, the resulting intermediates are readily oxidized by molecular oxygen to afford the amino‐substituted azothiazole derivatives. The latter exhibit remarkably red‐shifted absorption bands (*λ*_abs_=507–661 nm) with high molar extinction coefficients and show a strong positive solvatochromism. As revealed by spectrometric titrations and circular and linear dichroism studies, the water‐soluble, bis‐(dimethylaminopropylamino)‐substituted azo dye associates with duplex DNA by formation of aggregates along the phosphate backbone at high ligand–DNA ratios (LDR) and by intercalation at low LDR, which also leads to a significant increase of the otherwise low emission intensity at 671 nm.

## Graphical abstract

**Seeing red**: 5‐Monoamino‐ and 5,5′‐bisamino‐substituted azothiazole derivatives exhibit remarkably red‐shifted absorption bands depending on the substituents and the solvent (*λ*_abs_ = 507 – 661 nm). The water‐soluble, bis(dimethylaminopropylamino)‐substituted dye intercalates into duplex DNA, which leads to a significant increase of the emission intensity at 671 nm.

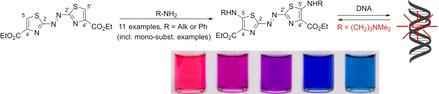

## Introduction

**Figure 1:**
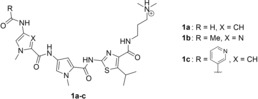
Structures of thiazole‐based DNA minor‐groove binders.

Aromatic azo compounds have been extensively investigated with regard to their photophysical and photochromic properties since they are commonly utilized in several different fields.^1^ For example, they are used in solar cells^2^ and solar thermal fuels,^3^ sensors,^4^ photopharmacology,^5^ and biomedical applications.^6^ Furthermore, they have been incorporated as molecular switches into polymers^7^ and carbohydrates^8^ as well as into biological systems^9^ such as oligonucleotides,^10^ peptides, and proteins.^11^ And azo derivatives have also been used in the design of (photoswitchable) ligands for duplex^12^ and quadruplex DNA.^13^ Most importantly, azo dyes represent the largest group of colorants with respect to their number and production volume in the chemical industry, mainly because they generally exhibit high molar extinction coefficients and colorfastness.^14^ Moreover, the most commonly employed synthesis of azo dyes by diazotization and azo coupling is not just straightforward but offers a huge structural diversity.^14^, ^15^ It is well known that the introduction of different electron‐donating and electron‐withdrawing substituents into the structure of azobenzene enables a significant variation of color.^14^ In this regard, the replacement of one or even both phenyl substituents by a heterocyclic unit, for example, pyridines, indoles, purines, thiophenes or various azoles, has recently gained great attention in order to further fine‐tune the optical and photochromic properties for different applications.^16^ Notably, the development of disperse azo dyes that are synthesized from heteroaromatic diazonium or heteroaromatic coupling components has already started over 30 years ago^17^ and led, for example, to the replacement of red and blue anthraquinone dyes.^18^ Thiazoles in particular have often been employed for the design of hetaryl‐substituted azo derivatives, and the photophysical properties and colorfastness of those azo dyes have been studied frequently.^19^ Specifically, the integration of a thiazole unit may lead to biologically active azo compounds, as thiazoles possess great biological relevance. For example, one of the most important compounds comprising a thiazole subunit is thiamine (vitamin B_1_).^20^ Thiazoles also occur in several other natural products such as cyclopeptides, which have shown cytotoxic activity^21^ or antibiotic properties.^22^ It was found, too, that thiazole polyamides, such as thiazotropsin A (**1 a**) and thiazotropsin B (**1 b**) as well as their analogues (e.g. **1 c**), bind to the minor groove of DNA (Figure 1).^23^ Furthermore, some thiazole‐based derivatives exhibit antiproliferative activity against selected human cancer cell lines^24^ and antibacterial activity.^25^

**Figure 2:**
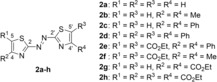
Structures of symmetrical azothiazole derivatives **2 a**–**h**.

In general, thiazoles are synthesized by the Hantzsch reaction, which is the condensation of an α‐halocarbonyl with a primary thioamide.^26^ By replacing the thioamide with bisthiourea hydrazothiazoles are available, which may be further oxidized by nitrous or nitric acid to afford the symmetric azothiazole derivatives **2 a**–**h** (Figure 2).^27^

In our search for novel, potentially photoswitchable DNA‐binding ligands,^12^, ^13^ we identified the ester‐substituted azothiazole derivatives **2 e**–**h** as promising platform for the synthesis of polyamides with DNA‐binding properties. These substrates are easily accessible and, even more importantly, the two identical thiazole substituents would allow a simultaneous modification of the ester groups on both sides of the azo unit. Remarkably, the corresponding azothiazole carboxylic acids, which would serve as useful intermediates for further derivatization, have not been reported, so far. In fact, attempts of their synthesis by saponification of **2 e**–**h** failed.27c, 27d Based on these reports, we tried to perform a direct amidation of the ester groups by the reaction with amines; however, in our attempt to functionalize the 4,4′‐substituted derivative **2 g**, we surprisingly discovered that the reaction of these substrates led to the formation of strongly colored 5‐alkylamino‐ or 5‐phenylamino‐substituted azothiazoles instead. As this appeared to be an efficient access to novel amino‐substituted azothiazole derivatives with a pronounced red‐shifted absorption from readily available substrates, we investigated this reaction in more detail. And herein, we present the scope and limits of this synthetic approach, and we demonstrate that the resulting 5‐alkyl‐ and 5‐phenylamino‐substituted azothiazoles have favorable absorption and DNA‐binding properties.

## Results

### Synthesis

**Scheme 1:**
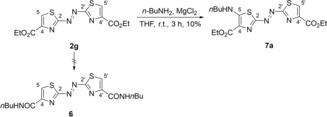
Synthesis of the 5‐butylamino‐substituted azothiazole **7 a**.

The known azothiazole diethyl ester **2 g** was synthesized by condensation of 2,5‐dithiobiurea (**3**) with ethyl 3‐bromopyruvate followed by oxidation of the intermediate hydrazothiazole **4** according to literature procedure (see Supporting Information).27d The addition of *n‐*butylamine (**5 a**) to a suspension of **1** and MgCl_2_ as Lewis acid in THF (Scheme 1)^28^ resulted in an immediate color change of the suspension from orange to magenta indicating a drastic bathochromic shift of the absorption maximum caused by significant structural changes in the conjugated aromatic system. The starting material **2 g** slowly dissolved and—as indicated by TLC control—was consumed after 3 h of stirring. Furthermore, a new intense magenta‐ and a faint purple‐colored spot were detected during TLC analysis (SiO_2_, *n*‐hexane/EtOAc 7:3, *R*_f_=0.31 and 0.44). The magenta‐colored main product was isolated by column chromatography (Table 1, entry 1). The NMR‐spectroscopic and the mass‐spectrometric analysis revealed that the ester functionalities were intact and that the amide **6** was not formed. Instead, the azothiazole **2 g** underwent formally a substitution in 5‐position resulting in the formation of the 5‐butylamino‐substituted azothiazole **7 a**. This unexpected finding led us to investigate the scope of the reaction further. We already suspected that the purple‐colored spot corresponded to the bis‐substituted product **8 a** and, in fact, prolongation of the reaction time and the use of five molar equivalents (equiv.) of *n‐*butylamine (**5 a**) resulted in the formation of **8 a** along with merely trace amounts of **7 a** (Table 1, entry 3).

**Table 1:** Synthesis of the mono‐ and bis‐substituted Azothiazoles **7 a**–**d** and **8 a**–**e**.

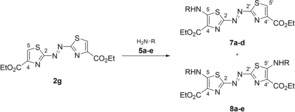							
Entry	Solvent	Amine	Molar	*t*	R	Yield [%]	
			equiv			**7**	**8**
1	THF	**5 a**	3	3 h	**a**:(CH_2_)_3_CH_3_	10	–^[b]^
2	CHCl_3_	**5 a** ^[a]^	1.5	24 h	**a**:(CH_2_)_3_CH_3_	21	0^[c]^
3	THF	**5 a**	5	12 d	**a**:(CH_2_)_3_CH_3_	–^[b]^	28
4	CHCl_3_	**5 b** ^[a]^	1.5	2 h	**b**:(CH_2_)_3_NMe_2_	42	0^[c]^
5	CHCl_3_	**5 b**	1.5	2 h	**b**:(CH_2_)_3_NMe_2_	50	0^[c]^
6	THF	**5 b**	4	9 d	**b**:(CH_2_)_3_NMe_2_	–^[b]^	38
7	CHCl_3_	**5 c**	4	>14 d	**c**:(CH_2_)_3_NHBoc	16	0^[c]^
8	THF	**5 c**	6	11 d	**c**:(CH_2_)_3_NHBoc	–^[d]^	–^[d]^
9	MeCN	**5 c**	4	5 h	**c**:(CH_2_)_3_NHBoc	12	–^[b]^
10	MeCN	**5 c**	4	19 h	**c**:(CH_2_)_3_NHBoc	–^[b]^	5
11	MeCN	**5 c** ^[a]^	3	16 h	**c**:(CH_2_)_3_NHBoc	–^[b]^	35
12	MeCN	**5 d** ^[a]^	3	7 d	**d**:4‐C_6_H_4_CH_3_	18	3
13	MeCN	**5 e** ^[a]^	8	7 d	**e**:4‐C_6_H_4_NMe_2_	–^[b]^	1

[a] With DABCO (1–2 molar equiv) as base. [b] Product was not isolated. [c]  Product was not formed according to TLC analysis. [d] The obtained mixture of mono‐ and bis‐substituted product (ratio ca. 25:75) could not be separated.

Similarly, the reaction of **2 g** with *N*,*N′*‐dimethyl‐1,3‐propanediamine (**5 b**), that was chosen because a dimethylamino group potentially increases water solubility, gave the respective bis‐substituted azothiazole **8 b** (entry 6). In order to allow further functionalization of the addition product we also wanted to introduce an alkyl chain bearing a terminal amino group. To avoid possible intermolecular side reactions, **2 g** was treated with the mono‐Boc‐protected propane‐1,3‐diamine (**5 c**). However, even with relatively long reaction times (11 d) and 6 molar equivalents of the amine (entry 8), the conversion to the bis‐substituted product **8 c** was very slow. And it turned out to be tedious work to separate the mono‐ and bis‐substituted products **7 c** and **8 c** by column chromatography with conventional solvent systems (Δ*R*_f_<0.1). Hence, in order to simplify purification and increase yields, the reaction conditions were optimized such that ideally either the respective mono‐ or the bis‐substituted azothiazole are formed exclusively. Firstly, the solvent was changed from THF to MeCN. Depending on the reaction time, the mono‐substituted product **7 c** and the bis‐substituted **8 c** were isolated after 5 and 19 h in 12 and 5 % yield, respectively (entries 9, 10). Remarkably, the addition of 2 equivalents of 1,4‐diazabicyclo[2.2.2]octane (DABCO) as an additional base led to a significant increase in the yield of **8 c** to 35 % in a much shorter reaction time (entry 11).

In CHCl_3_ as the solvent, the bis‐substituted azothiazole **8 c** was not formed even after 120 d of stirring, and the mono‐substituted dye **7 c** was isolated as the only addition product in 16 % yield (entry 7). Likewise, the reaction with the primary amines **5 a** and **5 b** in CHCl_3_ gave the mono‐substituted derivatives **7 a** and **7 b** as the only isolated addition products (entries 2, 5). Interestingly, in the case of **7 b**, the yield was slightly lower in the presence of one equivalent of DABCO (entry 4). The reaction of substrate **2 g** with the aromatic amines *p*‐toluidine (**5 d**) and *p*‐(dimethylamino)aniline (**5 e**) was also attempted to obtain the respective 5‐phenylamino‐substituted derivatives. While the reaction with **5 d** under the optimized reaction conditions in MeCN with DABCO as base gave the products **7 d** and **8 d** in 18 and 3 % yield, respectively, the derivatives **7 e** and **8 e** were hardly available by this method (entries 12, 13). Even with an excess of **5 e** (8 molar equivalents), we were only able to isolate the bis‐substituted product **8 e** in a yield of 1 %.

**Scheme 2:**
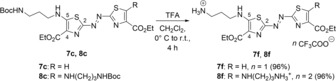
Synthesis of the 5‐ammoniumpropylamino‐substituted azothiazole derivatives **7 f** and **8 f**.

The Boc‐protected azothiazole derivatives **7 c** and **8 c** were deprotected with trifluoroacetic acid (TFA) to give the respective products **7 f** and **8 f** quantitatively as trifluoroacetate salts (Scheme 2). The structures of the new compounds **7 a**–**d**, **7 f** and **8 a‐**‐**f** were confirmed by NMR spectroscopy (^1^H, ^13^C, COSY, HSQC, HMBC) and electrospray ionization high‐resolution mass spectrometry (ESI‐HRMS).

### Absorption properties

**Figure 3:**
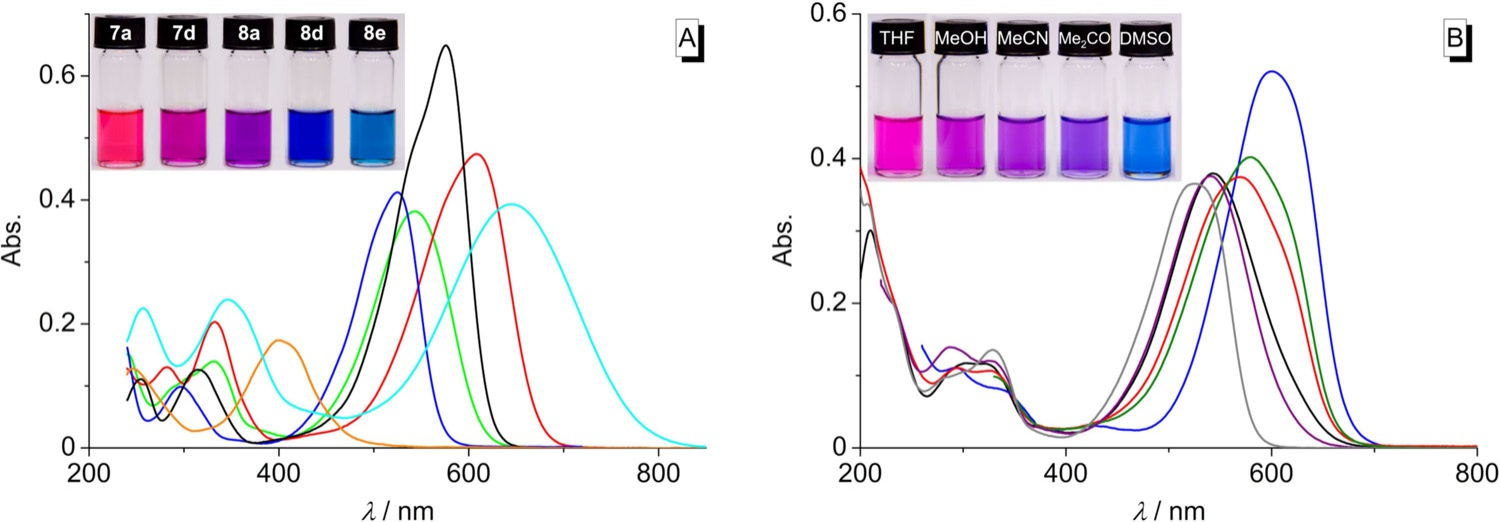
A: Color (*c=*50 μm) and absorption spectra (*c=*10 μm) of the derivatives **2 g** (orange), **7 a** (blue), **7 d** (green), **8 a** (black), **8 d** (red), and **8 e** (cyan) in CHCl_3_. B: Color (*c=*20 μm) and absorption spectra (*c=*10 μm) of derivative **7 d** in *n*‐hexane (gray), THF (purple), MeOH (black), MeCN (red), acetone (olive), and DMSO (blue).

The absorption properties of the azothiazole derivatives **2 g**, **7 a‐**‐**d**, **7 f**, and **8 a‐**‐**f** were investigated in representative nonpolar (*n*‐hexane, CHCl_3_), polar aprotic (THF, acetone, MeCN, DMSO) and polar protic solvents (H_2_O, MeOH, EtOH) (Table 2, Supporting Information Table S1). The solubility of the parent azothiazole **2 g** is somewhat limited, and the shift of its long‐wavelength absorption maximum is essentially independent from the solvent and just ranges from 397 nm in MeCN to 401 nm in THF (*ϵ*_max_=16 300–20 000 m ^−1^ cm^−1^) (Figure S2 A). In contrast, the monoalkylamino‐substituted derivatives **7 a**–**c** are sufficiently soluble in the investigated solvents. Furthermore, these strongly magenta‐colored dyes show broad absorption maxima that are significantly red‐shifted in comparison to the parent azothiazole **2 g** (*λ*_abs_=507–577 nm, *ϵ*_max_=33 800–48 000 m ^−1^ cm^−1^) (Table 2, Figure S2 B–D). As a general trend, the absorption bands of **7 a‐**‐**c** are red‐shifted going from a nonpolar solvent (*n*‐hexane for **7 a** and **7 b**, CHCl_3_ for **7 c**) to DMSO. Remarkably, the absorption maxima of the derivative **7 b** with the terminal dimethylamino group are slightly red‐shifted (4–9 nm) in comparison to **7 a** or **7 c** in all solvents except DMSO (31–36 nm). The aforementioned trends also apply to the respective bisalkylamino‐substituted azothiazoles **8 a**–**c**, but the additional amino functionality generally causes a more pronounced bathochromic shift of the long‐wavelength absorption band (*λ*_abs_=556–600 nm) and higher extinction coefficients (*ϵ*_max_=48 700–71 700 m ^−1^ cm^−1^), so that these dyes are strongly purple‐colored in solution (Table 2, Figure S3 A–C, Figure 3 A). The monophenylamino‐substituted azothiazole **7 d** exhibits the strongest positive solvatochromism; namely, the absorption band is red‐shifted by 74 nm from *n*‐hexane (*λ*_abs_=526 nm) to DMSO (*λ*_abs_=600 nm) (Figure 3 B, S2 E). The spectra of the bisphenylamino‐substituted dyes **8 d** and **8 e** show absorption bands that already lie in the red region of the visible spectrum (*λ*_abs_=600–619 nm for **8 d** and *λ*_abs_=626–661 nm for **8 e**) (Table 2, Figure S3 D–E). Unfortunately, their absorption properties in polar protic solvents and *n*‐hexane could not be determined due to the very low solubility in these solvents.

**Table 2:** Absorption Properties of the Azothiazole Derivatives **2 g**, **7 a**–**d** and **8 a**–**e**.

	**2 g**		**7 a**		**7 b**		**7 c**		**7 d**		**8 a**		**8 b**		**8 c**		**8 d**		**8 e**	
Solvent^[a]^	*λ*_abs_^[b]^	log *ϵ* ^[c]^	*λ*_abs_^[b]^	log *ϵ* ^[c]^	*λ*_abs_^[b]^	log *ϵ* ^[c]^	*λ*_abs_^[b]^	log *ϵ* ^[c]^	*λ*_abs_^[b]^	log *ϵ* ^[c]^	*λ*_abs_^[b]^	log *ϵ* ^[c]^	*λ*_abs_^[b]^	log *ϵ* ^[c]^	*λ*_abs_^[b]^	log *ϵ* ^[c]^	*λ*_abs_^[b]^	log *ϵ* ^[c]^	*λ*_abs_^[b]^	log *ϵ* ^[c]^
H_2_O	–^[d]^	–^[d]^	–^[d]^	–^[d]^	532	4.49	–^[d]^	–^[d]^	–^[d]^	–^[d]^	–^[d]^	–^[d]^	579	4.41	–^[d]^	–^[d]^	–^[d]^	–^[d]^	–^[d]^	–^[d]^
MeOH	–^[d]^	–^[d]^	529	4.66	537	4.59	528	4.65	542	4.58	580	4.84	582	4.81	580	4.78	–^[d]^	–^[d]^	–^[d]^	–^[d]^
EtOH	398	4.21	527	4.64	536	4.61	528	4.64	561	4.54	580	4.83	583	4.77	579	4.79	–^[d]^	–^[d]^	–^[d]^	–^[d]^
MeCN	397	4.30	531	4.61	540	4.61	531	4.61	567	4.57	575	4.83	580	4.82	575	4.77	–^[d]^	–^[d]^	–^[d]^	–^[d]^
DMSO	–^[d]^	–^[d]^	546	4.65	577	4.68	541	4.64	600	4.72	595	4.86	600	4.75	594	4.79	619	4.65	661	4.56
acetone	400	4.27	529	4.64	538	4.59	529	4.64	581	4.61	575	4.82	581	4.72	576	4.78	605	4.65	640	4.62
CHCl_3_	401	4.24	524	4.62	531	4.60	524	4.61	544	4.58	576	4.81	579	4.79	574	4.76	608	4.68	646	4.59
THF	401	4.23	525	4.61	530	4.61	525	4.57	540	4.58	574	4.80	578	4.74	575	4.75	600	4.71	626	4.61
*n*‐hexane	–^[d]^	–^[d]^	507	4.56	514	4.53	–^[d]^	–^[d]^	526	4.56	556	4.77	562	4.69	–^[d]^	–^[d]^	–^[d]^	–^[d]^	–^[d]^	–^[d]^

[a] Solvents arranged in order of decreasing *E*_T_^30^ values. [b] Long‐wavelength absorption maximum in nm; *c=*10 μm. [c] Molar extinction coefficient in cm^−1^ m ^−1^. [d] Not (fully) soluble.

### DNA‐binding properties

**Figure 4:**
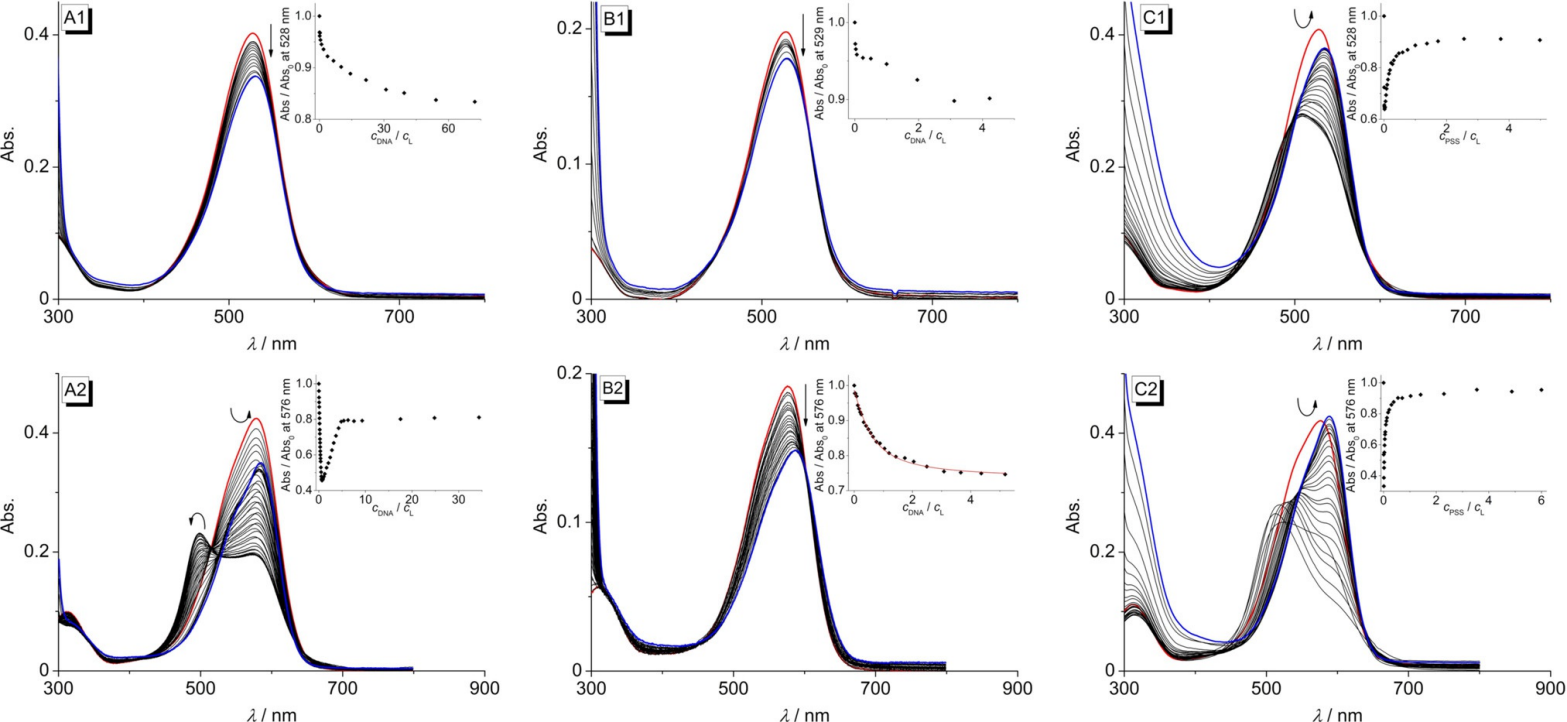
Spectrophotometric titration of **7 b** (1) and **8 b** (2) with ct DNA [A, *c*_L_=10 μm, *c*_DNA_=2.17 mm (A1), *c*_DNA_=1.49 mm (A2); *c*_DNA_ in base pairs] in BPE buffer (*c*_Na+_=16 mm, pH 7.0; with 5 % v/v DMSO), **22AG** (B, *c*_L_=5 μm, *c*_**22AG**_=285 μm; *c*_**22AG**_ in oligonucleotide) in K‐phosphate buffer pH 7.0 (*c*_K+_=73 mm; with 5 % v/v DMSO) and PSS [C, *c*_L_=10 μm, *c*_PSS_=190 μm (C1), *c*_PSS_=195 μm (C2)] in BPE buffer (*c*_Na+_=16 mm, pH 7.0; with 2.5–5 % v/v DMSO). Red: Spectra of the pure ligand solutions; blue: spectra at the end of the titrations. The arrows indicate the changes of absorption upon addition of the host molecule. Insets: Plot of Abs./ Abs._0_ versus *c*_DNA_/*c*_L_ or *c*_PSS_/*c*_L_.

The changes of the absorbance upon addition of double‐stranded calf thymus (ct) DNA to the azothiazoles **7 b**, **8 b**, and **8 f** were followed by spectrophotometric titrations (Figure 4, Figure S6, Table 3). The addition of ct DNA to a solution of **7 b** caused only a slight decrease of the absorbance and a small red shift (Δ*λ*=3 nm) (Figure 4 A1), whereas the addition of DNA to a solution of **8 b** also resulted in the formation of a distinct additional blue‐shifted band with a maximum at 499 nm along with the decrease of the initial absorbance and formation of an isosbestic point at 515 nm. At a smaller ligand‐DNA ratio (LDR) of <1.4 the blue‐shifted peak disappeared and the absorbance increased, so that eventually a red shift of Δ*λ*=9 nm of the maximum was observed (Figure 4 A2). The titration of ct DNA to **8 f** essentially resulted in the same course of the titration, that is, the formation of a blue‐shifted band located at 504 nm at LDR>0.8 and a subsequent increase in absorbance with a red shift of Δ*λ*=8 nm (Figure S6).

**Table 3:** Absorption and Emission Properties of **7 b** and **8 b** in the Presence of ct DNA, **22AG** and PSS.

	*λ*_abs_^[a]^	log *ϵ* ^[b]^	Δ*λ*_abs_^[c]^			*λ*_fl_^[d]^	Δ*λ*_fl_^[e]^		
			ct DNA	**22AG**	PSS		ct DNA	**22AG**	PSS
**7 b**	528	4.60	3	0	7	–^[f]^	–^[f]^	–^[f]^	–^[f]^
**8 b**	576	4.61	9	12	13	671	−21	−21	−22

[a] Long‐wavelength absorption maximum (in nm); *c=*5–10 μm in phosphate buffer (pH 7.0; with 2.5–5 % v/v DMSO). [b] Molar extinction coefficient. [c] Shift of the long‐wavelength absorption maximum between free and bound ligand (in nm). [d] Long‐wavelength emission maximum (in nm); *c=*5–10 μm in phosphate buffer (with 2.5–5 % v/v DMSO), *λ*_ex_=515 nm. [e] Shift of the long‐wavelength emission maximum between free and bound ligand (in nm). [f] Not determined.

Additionally, the interactions of the dimethylaminopropylamino‐substituted derivatives **7 b** and **8 b** with the quadruplex‐forming oligonucleotide d[A(GGGTAA)_3_GGG] (**22AG**) as well as with polystyrene sulfonate (PSS) were studied. While the titration of **22AG** to a solution of **7 b** only led to a very small decrease of the absorbance (Figure 4 B1), the addition to **8 b** also resulted in a pronounced red shift of the absorption maximum (Δ*λ*=12 nm) (Figure 4 B2). The results of the photometric DNA titrations were analyzed according to the established protocol,^29^ but the fitting of the binding isotherms to the theoretical model was only possible for titration of **8 b** with **22AG** (*K*_**22AG**_=2.4×10^4^, *n=*2.1) (Inset in Figure C2). On titration of PSS to **7 b** and **8 b**, the absorbance at 528 nm (**7 b**) and 576 nm (**8 b**) decreased until ligand‐PSS ratios of >60 (**7 b**) and >115 (**8 b**) were reached (Figure 4 C1 and C2). This hypochromic effect was accompanied by a hypsochromic shift of the absorption band to 508 nm (**7 b**) and 513 nm (**8 b**). In both cases, the absorption increased at lower ligand‐PSS ratios, and when saturation was reached the absorption maxima were eventually red‐shifted by 7 nm and 13 nm, respectively, in comparison to the pure ligand solutions.

**Figure 5:**
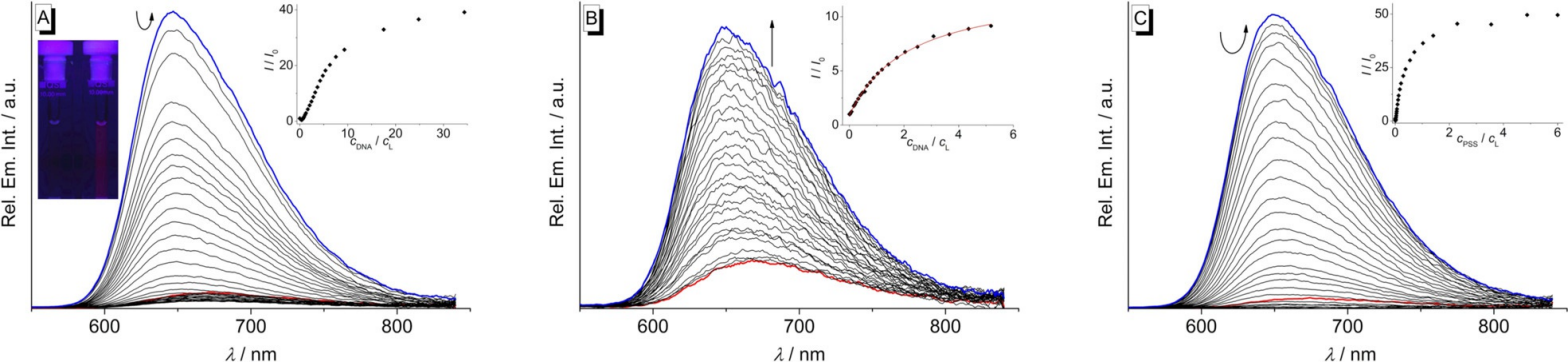
Spectrofluorimetric titration of **8 b** with ct DNA (A, *c*_L_=10 μm, *c*_DNA_=1.49 mm; *c*_DNA_ in base pairs) in BPE buffer (*c*_Na+_=16 mm, pH 7.0; with 5 % v/v DMSO), **22AG** (B, *c*_L_=5 μm, *c*_**22AG**_=285 μm; *c*_**22AG**_ in oligonucleotide) in K‐phosphate buffer (*c*_K+_=73 mm, pH 7.0; with 5 % v/v DMSO) and PSS (C, *c*_L_=10 μm, *c*_PSS_=195 μm) in BPE buffer (*c*_Na+_=16 mm, pH 7.0; with 2.5 % v/v DMSO); *λ*_ex_=515 nm. Red: Spectra of the pure ligand solutions; blue: spectra at the end of the titrations. The arrows indicate the changes in emission intensity upon addition of the host molecule. Insets: Plot of the relative fluorescence intensity *I*/*I*_0_ (corrected with regard to the change of the absorption at the excitation wavelength) versus *c*_DNA_/*c*_L_ or *c*_PSS_/*c*_L_. Inset pictures in A: Fluorescence colors of **8 b** in the absence and in the presence of ct DNA; *λ*_ex_=366 nm. The contrast and brightness were enhanced by 30 % without changing the true colors (cf. Figure S5).

Upon excitation at *λ*_ex_=515 nm the derivative **7 b** is essentially non‐fluorescent both in the absence or presence of DNA or PSS. The azothiazole **8 b**, however, exhibits a broad emission band with very low fluorescence intensity (*Φ*_fl_=0.002 relative to cresyl violet^30^) at 671 nm. The addition of ct DNA to **8 b** led initially to a further decrease of the fluorescence intensity with increasing DNA concentration until an LDR of >3 was reached (Figure 5 A). At lower LDR values the development of a new blue‐shifted emission band was observed (*I*/*I*_0_=39, *Φ*_fl_=0.029) (Table 3). The addition of **22AG** to **8 b** resulted in the formation of essentially the same blue‐shifted emission band (Figure 5B) with a smaller light‐up effect (*I*/*I*_0_=9). On titration of PSS to **8 b** the increase of the fluorescence intensity at low ligand‐PSS ratios of <8 was, however, slightly more pronounced (*I*/*I*_0_=50) (Figure 5 C) as compared to addition of ct DNA. Remarkably, a solution of **8 b** in a highly viscous medium such as glycerol also exhibited a broad fluorescence band at *λ*_fl_=655 nm (*Φ*_fl_=0.10) (Figure S1).

**Figure 6:**
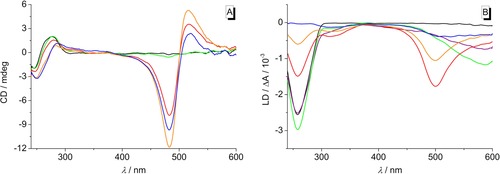
CD spectra (A, *c=*10 μm) and LD spectra (B, *c=*20 μm) of ct DNA in the absence and presence of **8 b** at LDR=0 (black), 0.20 (purple, omitted in A), 0.50 (green), 1.00 (red), 1.50 (orange), 2.00 (blue) in BPE buffer (*c*_Na+_=16 mm, pH 7.0; with 5 % v/v DMSO).

In order to examine the binding mode of **8 b** with DNA, circular dichroism (CD) and linear dichroism (LD) studies with ct DNA were performed (Figure 6). At small LDR values of 0.2 and 0.5 no apparent induced circular dichroism (ICD) band was detected (Figure 6 A), but a broad negative LD signal at around 585 nm appeared (Figure 6 B). At an LDR>0.5 an intense ICD band at 410–590 nm with a bisignate shape and an isoelliptic point at 500 nm as well as a negative LD signal at 500 nm developed, while the latter signal decreased in intensity from LDR=1 to 2. Notably, the intensity of the negative LD signal of the DNA at 258 nm decreased and eventually vanished at LDR=2.

## Discussion

### Synthesis

**Scheme 3:**
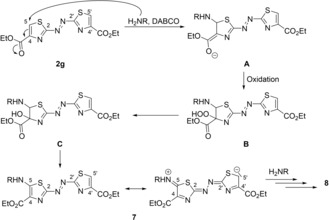
Proposed mechanism for the formation of **8** (cf. ref. 33, 34).

Our attempts to perform an amidation of the azothiazole diester **2 g** led to the discovery of novel 5‐alkylamino‐ and 5‐phenylamino‐substituted azothiazole derivatives, that resulted from the initial nucleophilic addition of the respective amines at the C5 carbon atom. Usually, thiazoles readily react in electrophilic aromatic substitution reactions, if the thiazole ring is substituted with electron‐donating groups.^31^ For example, 2‐aminothiazole reacts with electrophiles such as bromine or diazonium salts at C5 to afford the respective 5‐bromo‐ and 5‐arylazo‐substituted thiazoles.19d, 19e, 26, 32 The ester functionality of azothiazole **2 g** at C4, however, significantly reduces the electron density of the thiazole ring. Therefore, it stands to reason that in the first step of the reaction mechanism, the thiazole undergoes a Michael‐type addition at C5, which is in β‐position to the ester (Scheme 3 A). This proposed mechanism is consistent with the observation that the reaction with a rather weak nucleophile such as *p*‐toluidine (**5 d**) gave lower yields of the addition products **7 d** and **8 d** (Table 1, entry 12) as compared to the reactions with the primary alkyl amines **5 a‐**‐**c**, even though longer reaction times were employed. The very low yield of **8 e** (Table 1, entry 13) is probably caused by slow oxidation of *p*‐(dimethylamino)aniline (**5 e**) under the employed aerobic conditions. During the optimization of the reaction conditions, we found that the addition of DABCO led to a significantly increased yield of derivative **8 c** (Table 1, entries 10 and 11). However, it should be noted that the substrate **2 g** also slowly decomposes in the presence of a base such as DABCO and therefore, the reaction has to be monitored carefully in order to avoid the formation of unidentified side products. Nevertheless, the presence of DABCO in the reaction mixture appears to be advantageous. In fact, it has been shown already that the oxidation of thiazoline 4‐carboxylate to thiazole 4‐carboxylates by molecular oxygen is supported by bases, presumably as the latter promotes the formation of the enolate **A** (Scheme 3), which is a prerequisite for the subsequent oxidation to **B**.^33^, ^34^ Hence, we propose that the α‐hydroxy thiazoline **C** is formed as an intermediate that finally undergoes elimination, supposedly also assisted by DABCO, to achieve aromatization^20^ and to form the monoamino‐substituted thiazole **7**. The electron‐donating 5‐amino‐substituent, however, reduces the reactivity of the mono‐substituted azothiazoles so that these intermediates are less susceptible towards a second nucleophilic addition at C5′, as shown by the respective resonance structure of **7** (Scheme 3). This particular electron distribution results in a lower reaction rate and therefore longer reaction times are required to obtain the bis‐substituted products (Table 1, entries 3 and 6). Remarkably, the monoalkylamino‐substituted azothiazoles **7 a‐**‐**c** were isolated as the only addition products (Table 1, entries 2, 4, 5, 7), when the reactions of azothiazole **2 g** with the alkylamines **5 a**–**c** were carried out in CHCl_3_, while the use of an aprotic, polar solvent such as THF or MeCN also afforded the respective bis‐substituted derivatives. We assume that in contrast to the nonpolar CHCl_3_ the latter solvents have a stabilizing effect on the charged intermediates that are formed during the second nucleophilic addition. In this regard, the use of two different solvents, that is, CHCl_3_ and MeCN, in separate subsequent reaction steps may even allow the introduction of two different alkyl‐ or phenylamino substituents in order to further tune the absorption properties.

### Absorption properties

The introduction of amino substituents as strong electron‐donating groups into the azothiazole structure generally enhances the delocalization of the conjugated system and therefore causes a significant red shift of the long‐wavelength absorption maximum in comparison to the parent compound **2 g**. Accordingly, the most pronounced red shift was observed for azothiazole **8 f** (*λ*_abs_=626–661 nm) bearing two *p*‐(dimethylaminophenyl)amino substituents as the strongest electron donors. As a general trend, the derivatives **7 a**–**d** and **8 a**–**c** exhibit positive solvatochromism with the longest wavelength absorption band in DMSO and the shortest in a nonpolar solvent (*n*‐hexane or CHCl_3_). This characteristic behavior may originate either from the stabilization of the Franck–Condon excited state or from the destabilization of the ground state with increasing solvent polarity. However, donor‐acceptor‐substituted azo dyes have a higher dipole moment in the excited state than in the ground state, and therefore, the excited state is better stabilized by solvation in a polar solvent.^35^ With a strong donor substituent on just one side of the azo unit, the 5‐(*p*‐toluidylamino)‐substituted derivative **7 d** has the highest dipole moment and therefore exhibits the strongest solvatochromism of all derivatives (Figure 3 B).

**Figure 7:**
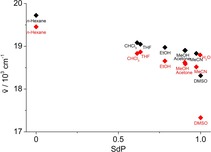
Plot of the absorption maximum of **7 a** (black) and **7 b** (red) in the respective solvents versus the dipolarity SDP.38a

To analyze the solvent‐dependent shift of the absorption maximum several common solvatochromic empirical parameters describing nonspecific or specific solute–solvent interactions were employed.^35^, ^36^ Among those parameters are the widely used Kamlet–Taft parameters, namely the hydrogen‐bond‐donating ability (*α*), the hydrogen‐bond‐accepting ability (*β*) and the dipolarity/polarizability polarity (π*),^37^ and the more recently developed Catalán parameters, namely the solvent acidity (SA), the solvent basicity (SB), the solvent polarizability (SP) and the solvent dipolarity (SdP).^38^ Thereby, the latter empirical scales by Catalán et al. are advantageous in so far as they are based on defined reference processes and provide an independent polarizability and dipolarity scale. Overall, we found that the solvatochromism of the azothiazoles **7 a**–**c** is mainly affected by the dipolarity of the solvent. Specifically, the plots of the absorption maxima correlate well with the SdP scale (**7 a**: *r* ^2^=0.98, **7 b**: *r* ^2^=0.99, **7 c**: *r* ^2^=0.92), at least if DMSO and H_2_O are excluded as solvents (Figure 7, S4 A). This analysis indicates that an increasing dipolarity of the solvent leads to a more pronounced stabilization of the excited molecule thus leading to a red‐shifted absorption.

The effect that the absorption spectra in DMSO are shifted to lower wavenumbers to a much greater extent was previously attributed to the high propensity of DMSO to act as hydrogen‐bond acceptor resulting in the formation of hydrogen‐bonding complexes that cause an even more pronounced stabilization of the excited state.35c, 35d In contrast to the alkylamino‐substituted azothiazoles **7 a‐**‐**c**, the absorption maxima of **7 d** and **8 a‐**‐**c** do not correlate well with the dipolarity (Figure S4 B–E) or any other particular solvent property. Apparently, several solvent properties contribute simultaneously, but to different extent to the stabilization of the excited state of these derivatives which in turn influences the shift of the absorption bands.

The longest wavelength absorption maximum of the bisphenylamino‐substituted derivatives **8 d** und **8 e** was observed in DMSO, which indicates a strong stabilization of theses dyes in the excited state by this solvent, too (Figure S3 D, E). Unfortunately, the solvatochromism could not be investigated in more detail because of the limited solubility of these derivatives, especially in protic solvents.

### DNA‐binding properties

The water‐soluble derivatives **7 b**, **8 b**, and **8 f** were also investigated regarding their DNA‐binding properties. Firstly, the course of the spectrophotometric titrations revealed that all three azothiazoles associate with DNA; however, the addition of ct DNA or the quadruplex‐forming oligonucleotide **22AG** to the monoamino‐substituted azothiazole **7 b** only caused minor changes in the absorption spectra indicating a weak binding affinity to both forms of DNA (Figure 4 A1, B1). In contrast, the titrations of DNA to the bis‐substituted azothiazoles **8 b** and **8 f** revealed that these dyes have at least a strong binding affinity to ct DNA with different binding modes at varying LDR (Figure 4 B2, S6). Specifically, the development of a distinct blue‐shifted band at higher LDR, that is, at high dye loading at the DNA, implied that these dyes at first form aggregates along the phosphate backbone of the DNA (“outside edge binding”) due to the lack of available binding sites. This binding mode was confirmed by the additional CD‐spectroscopic studies performed with derivative **8 b** since the bisignate‐shape of the ICD bands at around 410–590 nm as well as the zero transition of this band at the absorption maximum of the bound dye clearly indicate exciton coupling between the stacked chromophores (Figure 6 A).^39^ The respective negative LD signal at the same wavelength points towards the conclusion that these aggregates are oriented with the aromatic plane of the dyes perpendicular to the DNA helix (Figure 6 B). Remarkably, the negative LD signal of the DNA is successively diminished at LDR>0.5, which implies that the DNA is significantly bent or compacted due to the formation of the dye aggregates and therefore becomes less oriented by the shear flow.^40^

**Figure 8:**
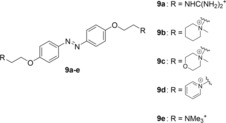
Structures of the DNA‐binding azobenzene derivatives **9 a**–**e**.

With an increasing number of binding sites available at lower LDR, the azothiazoles **8 b** and **8 f** have a more specific binding to the DNA which is supported by the increase in absorption accompanied by a significant red shift.^41^ Thereby, the negative LD signal at 585 nm, coinciding with the absorption maximum of the red‐shifted band of the ligand‐DNA complex, shows that **8 b** intercalates into the DNA at this LDR,^42^ which is in agreement with previous DNA binding studies of the guanidinium‐substituted azobenzene derivative **9 a**.12a The corresponding reduced linear dichroism (LD^r^) spectra provide additional information about the average orientation of the ligand transition dipole moment relative to the DNA base transitions (Figure S7). At an LDR=0.2, the LD^r^ values at around 585 nm are smaller than at 260 nm, thus indicating that the transition moment of the dye **8 b** is tilted relative to the plane of the DNA base pairs by approximately 24°. Moreover, the terminal, positively charged protonated amino substituents of **8 b** and **8 f** may associate with the minor groove causing an additional stabilization of the ligand‐DNA complex.^43^ The spectrophotometric titration of **22AG** to **8 b** (Figure 4 B2) revealed that this derivative also binds to quadruplex DNA, however with low binding affinity (*K*_**22AG**_=2.4×10^4^). The continuous decrease of the absorption accompanied by a small red shift upon addition of **22AG** indicates that **8 b** does not form aggregates in the presence of **22AG** and binds mostly with one binding mode to quadruplex DNA, namely by terminal π‐stacking as was shown for the structurally similar azobenzene derivatives **9 b**–**e** (Figure 8).^13^

The aggregation behavior of the azothiazole derivatives **7 b** and **8 b** was investigated further by spectrometric titrations of PSS—as a representative, anionic polyelectrolyte—to aqueous solutions of **7 b** and **8 b** (Figure 4 C1, C2). The initial decrease in absorption and the hypsochromic shift of the respective absorption bands indicated the formation of H‐aggregates at low PSS concentrations.^44^ Similarly to the titration with DNA, a further increase of the PSS concentration again led to a bathochromic shift of the absorption band due to electrostatic interactions of the azothiazoles **7 b** and **8 b** with PSS on well‐separated binding sites.^45^

In the absence of DNA or PSS, the derivative **8 b** is only weakly fluorescent in aqueous solution because of radiationless deactivation of the excited state, presumably by *E–Z* isomerization of the azo double bond which is in turn followed by a very fast thermal *Z*–*E* isomerization.1a, 16a, 16g At low ct DNA or PSS concentrations, the formation of aggregates (see above) initially leads to self‐quenching, and a further decrease of the already low fluorescence intensity was detected (Figure 5 A, C).^45^, ^46^ Accordingly, this behavior was not observed upon addition of **22AG** (Figure 5 B) because **8 b** does not form aggregates in the presence of quadruplex DNA. When binding in a more specific manner to ct DNA or PSS at a higher concentration of the respective host system, **8 b** experiences a significant increase in fluorescence intensity, which can also be seen by the naked eye under UV light (Inset in Figure 5 A). Considering that this light‐up effect takes place only as soon as a particular ligand–DNA ratio is adjusted (Figure 5 A), the limiting value for this fluorimetric detection is *c*_DNA_=7 μm. Since **8 b** exhibits the same emission in a highly viscous solvent such as glycerol (Figure S1), it may be concluded that the conformational flexibility within the binding site is reduced leading to suppression of the radiationless deactivation pathways of the excited state.^47^ Similar fluorescence light‐up effects in media with restricted free volume, that is, in the presence of DNA or in glycerol, were also observed for amino‐ and aminostyryl‐substituted quinolizinium derivatives.^48^ The fact that the fluorescence light‐up effect in the presence of **22AG** is comparatively less pronounced leads to the conclusion that binding of **8 b** to the quadruplex DNA by terminal π‐stacking does not reduce its conformational flexibility as much as in the intercalation binding site in duplex DNA.

## Conclusions

The readily available diethyl azothiazole‐4,4′‐dicarboxylate (**2 g**) unexpectedly undergoes a Michael‐type addition with primary alkyl and aromatic amines at C5, and the intermediates are readily oxidized by molecular oxygen under aerobic conditions to afford novel mono‐ and bis‐substituted 5‐alkyl‐ and 5‐phenylamino azothiazole derivatives. While their synthesis admittedly requires further optimization to increase the yields, the aminopropylamino‐substituted azothiazoles **7 f** and **8 f** even offer the opportunity for further functionalization. In general, all azothiazoles exhibit not only high color strength, but also remarkably red‐shifted absorption bands in comparison to the parent compound **2 g**. In this regard, they also show positive solvatochromism with derivative **7 d** featuring the strongest bathochromic shift when changing from a nonpolar to a polar solvent. As revealed by spectrometric titrations, the water‐soluble dyes **7 b** and **8 b** associate with DNA, whereas the bis(dimethylaminopropylamino)‐substituted azothiazole **8 b** has a higher binding affinity to ct DNA and quadruplex DNA in comparison to the mono‐substituted derivative **7 b**. The additional CD and LD studies revealed that **8 b** forms chiral aggregates in the presence of ct DNA at high dye loading and intercalates into the DNA with ample availability of binding sites at lower dye loading. Thus, the derivative **8 b** represents one of the rare representatives of azo dye‐based DNA intercalators. Most notably, the association of **8 b** to DNA also leads to a significant increase of the low emission intensity at 671 nm, that is, in an advantageous range for biological applications, so that these novel azothiazoles may be considered as promising starting point for the development of DNA‐sensitive fluorescent dyes.

## Experimental Section

### Equipment

NMR spectra were recorded with a Bruker Avance 400 (^1^H: 400 MHz, ^13^C: 100 MHz) at room temperature (approximately 22 °C), with a Jeol ECZ 500 (^1^H: 500 MHz, ^13^C: 125 MHz) at 25 °C or with a Varian VNMR‐S 600 (^1^H: 600 MHz, ^13^C: 150 MHz) at 25 °C. Spectra were processed with the software MestReNova (version: 12.0.1) and referenced to the respective solvent ([D_6_]DMSO: *δ*_H_=2.50, *δ*_C_=39.5; CDCl_3_: *δ*_H_=7.27, *δ*_C_=77.0). The chemical shifts are given in ppm. Absorption spectra were recorded with a Cary 100 Bio or with an Analytik Jena Specord S 600 spectrophotometer in Hellma quartz cells 110‐QS or 114B‐QS (10 mm) with baseline correction at 20 °C. Emission spectra were collected with a Cary Eclipse spectrophotometer in Hellma quartz cells 114F‐QS (10 mm×4 mm) at 20 °C. Circular dichroism (CD) and linear dichroism (LD) spectra were measured with an Applied Photophysics Chirascan spectropolarimeter. For LD spectra the CD spectrometer was equipped with a High Shear Couette Cell Accessory. The LD samples were recorded in a rotating couette with a shear gradient of 1200 s^−1^. Mass spectra (ESI) were recorded on a Finnigan LCQ Deca (*U*=6 kV; working gas: Argon; auxiliary gas: Nitrogen; temperature of the capillary: 200 °C). High‐resolution mass spectra were acquired with a Thermo Fisher Scientific Exactive mass spectrometer with Orbitrap mass analyzer and the exact masses of the analyte ions were measured. For analyte ionization a heated electrospray ionization source (HESI‐II) in positive‐ion detection mode (*U*=4.5 kV) was used. Samples were introduced into the HESI‐II‐MS system via flow injection by an Agilent 1200 HPLC instrument (analyte concentration: 10 or 100 μm, injected sample volume: 10 μL, flow rate: 0.2 mL min^−1^, mobile phase: MeOH with 0.1 % formic acid). The melting points were measured with a BÜCHI 545 (BÜCHI, Flawil, CH) and are uncorrected.

### Materials

All commercially available chemicals were reagent‐grade and used without further purification unless otherwise mentioned. Spectroscopic grade solvents were used for solutions submitted to absorption and emission spectroscopy. All buffer solutions were prepared from purified water (resistivity 18 MΩ cm) and biochemistry‐grade chemicals. The buffer solutions were filtered through a PVDF membrane filter (pore size 0.45 μm) prior to use. The oligodeoxyribonucleotide **22AG** d[A(GGGTTA)_3_GGG] (purification: HPLC; quality control: MALDI‐TOF; synthesis scale: 1.0 μmol) was purchased from biomers.net GmbH (Ulm, Germany). Calf thymus DNA (type I; highly polymerized sodium salt; *ϵ*=12824 cm^−1^ m ^−1^) was purchased from Sigma–Aldrich. Poly(styrene sulfonic acid) (PSS, sodium salt, M.W. 70 000) was purchased from Alfa Aesar. BPE (biphosphate EDTA) buffer: 6.0 mm Na_2_HPO_4_, 2.0 mm NaH_2_PO_4_, 1.0 mm Na_2_EDTA, pH 7.0; potassium phosphate buffer: 25 mm K_2_HPO_4_, 70 mm KCl; adjusted with 25 mm KH_2_PO_4_ to pH 7.0.

### Methods

Solutions were prepared for each measurement from stock solutions in a suitable solvent (CHCl_3_ for **2 g**, **7 a**–**d**, **8 a**–**e**; MeOH for **7 f** and **8 f**; *c=*1.0 mm). For experiments in different solvents, aliquots of the stock solution were evaporated under a stream of nitrogen and redissolved in the respective solvent. In general, absorption spectra were determined in a range between 200 and 850 nm (260–850 nm for DMSO, 330–850 nm for acetone, 240–850 nm for CHCl_3_, and 220–850 nm for THF) and subsequently smoothed in the Origin software with the function “adjacent‐averaging” (factor of 10). For the detection of emission spectra the excitation and emission slits were adjusted to 5 nm, the detection speed was 120 nm min^−1^, and the detector voltage was adjusted between 550 and 700 V depending on the fluorescence intensity. The emission spectra were smoothed with the implemented moving‐average function by a factor of 5. Emission spectra in the range between 600 and 850 nm were corrected using an instrument specific correction curve. The fluorescence quantum yields of derivative **8 b** were determined relative to cresyl violet (*Φ*_fl_=0.54 in MeOH)30a according to the established procedures.30b, 30c

The spectrometric titrations were performed according to published protocols,48b and the binding constants were determined by fitting the binding isotherms to the established theoretical model according to the independent‐site model.^29^

For the CD and LD experiments six samples were prepared with a fixed ct DNA concentration (*c*_DNA_=10 or 20 μm). In five of the samples different amounts of ligand **8 b** were added to obtain ligand‐DNA ratios of 0.2, 0.5, 1.0, 1.5, 2.0. All samples contained 5 % v/v DMSO. CD and LD spectra were recorded in a range between 240 and 600 nm with a band width of 1 nm, a recording speed of 1 nm s^−1^ and a time per data point of 0.5 s. The reduced linear dichroism, LD^r^, and the angle *α* between the electric dipole moment of the ligand and the DNA helix axis were determined according to the established procedures.^42^, ^49^

## Conflict of interest

The authors declare no conflict of interest.

## Supporting information

As a service to our authors and readers, this journal provides supporting information supplied by the authors. Such materials are peer reviewed and may be re‐organized for online delivery, but are not copy‐edited or typeset. Technical support issues arising from supporting information (other than missing files) should be addressed to the authors.

Supplementary
