# Supplementary material for: Synthesis of 5‐Alkyl‐ and 5‐Phenylamino‐Substituted Azothiazole Dyes with Solvatochromic and DNA‐Binding Properties
Source: Chemistry. 2019 Nov 8;25(70):16088–98. doi: 10.1002/chem.201903657 (PMC6973281; doi:10.1002/chem.201903657)
Supplement: Supplementary file 1 — Supplementary [file CHEM-25-16088-s001.pdf]

CHEMISTRY
A European Journal

Supporting Information

Synthesis of 5-Alkyl- and 5-Phenylamino-Substituted Azothiazole Dyes with Solvatochromic and DNA-Binding Properties

Phil M. Pithan, Christopher Kuhlmann, Carsten Engelhard, and Heiko Ihmels*[a]

chem_201903657_sm_miscellaneous_information.pdf

Table of contents

1 Experimental Section **2**

1.1 Methods 2

1.1.1 Determination of fluorescence quantum yields of azothiazole **8b** 2

1.2 Synthesis 2

1.2.1 Synthesis of diethyl 2,2'-(diazene-1,2-diyl)-(*E*)-bis(thiazole-4-carboxylate) (**2g**) 2

1.2.2 Synthesis of the azothiazole derivatives **7a–f** and **8a–f** 3

2 Additional spectroscopic data **11**

2.1 Absorption and emission properties 11

2.2 DNA-binding properties 15

3 ^{1}H- and ^{13}C-NMR spectra **17**

4 References **30**

1 Experimental Section

1.1 Methods

Physical-chemical and essential preparative procedures were performed at least twice to check reproducibility.

*1.1.1 Determination of fluorescence quantum yields of azothiazole **8b***

The relative fluorescence quantum yields of the azothiazole derivative **8b** were determined under identical conditions, *i.e.* the same cuvettes were used and the measurements were performed at a constant temperature with the same settings on the spectrometer (detection wavelength, excitation wavelength, detector voltage, slit bandwidths, collection rate). Cresyl violet (Φ_{fl} = 0.54 in MeOH)[1] was used as standard. The emission spectra were collected from diluted solutions with Abs. = 0.10 at the excitation wavelength λ_{ex} = 515 nm. After integration of the fluorescence band, the relative fluorescence quantum yields were calculated according to Eq. 1.[2]

$$\phi_F = \frac{J_x \cdot (1 - T_S)}{J_S \cdot (1 - T_x)} \cdot \frac{n_x^2}{n_S^2} \cdot \phi_{F,S} \qquad \text{(Eq. 1)}$$

The subscripts “x” and “s” refer to the substance under investigation and a reference compound, respectively; $J = \int I_F(\lambda)d\lambda$ is the emission integral over the area of interest; T is the optical transmittance of the sample solution at the excitation wavelength, λ_{ex}; n is the refractive index of the sample or standard solution.

1.2 Synthesis

*1.2.1 Synthesis of diethyl 2,2'-(diazene-1,2-diyl)-(E)-bis(thiazole-4-carboxylate) (**2g**)*

The known azothiazole **2g** was synthesized according to the published procedure (Scheme S1).[3]

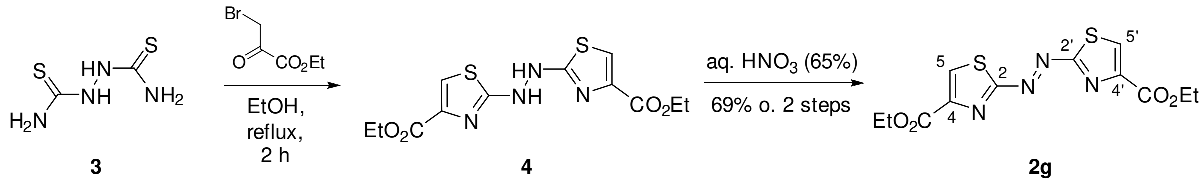

Scheme S1. Synthesis of azothiazole **2g**.[3]

Diethyl 2,2'-(hydrazine-1,2-diyl)-bis(thiazole-4-carboxylate) (**4**)

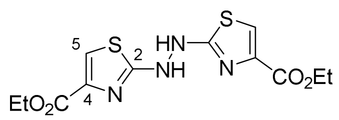

Under an argon atmosphere a solution of 2,5-dithiobiurea (**3**) (3.00 g, 20.0 mmol) and ethyl 3-bromopyruvate (7.80 g, 40.0 mmol, 6.69 mL, tech. 75%) in ketone-free EtOH (30 mL) was stirred for 2 h under reflux. The reaction mixture was cooled to 0 °C. The precipitate was filtered, washed with ice-cold MeOH (5 x 10 mL) and Et_2O (2 x 30 mL). The product **4** was obtained as light ochre-colored solid (5.30 g) and used without purification. A pure, white sample of the hydrazine **4** was obtained by crystallization from the mother liquor and washing with cold EtOH; mp > 220 °C (dec.) (lit.: 227–228 °C[3]). – ^{1}H-NMR (400 MHz, DMSO-d_6): δ = 1.27 (t, ^3J = 7 Hz, 6 H, 4-$CO_2CH_2\underline{CH_3}$), 4.23 (q, ^3J = 7 Hz, 4 H, 4-$CO_2\underline{CH_2}CH_3$), 7.75 (s, 2 H, 5-H), 10.26 (s, 2 H, NH). – ^{13}C-NMR (100 MHz, DMSO-d_6): δ = 14.2 (4-$CO_2CH_2\underline{CH_3}$), 60.4 (4-$CO_2\underline{CH_2}CH_3$), 119.3 (C5), 143.1 (C4), 160.8 (4-$\underline{C}O_2CH_2CH_3$), 172.4 (C2).

Diethyl 2,2'-(diazene-1,2-diyl)-(*E*)-bis(thiazole-4-carboxylate) (**2g**)

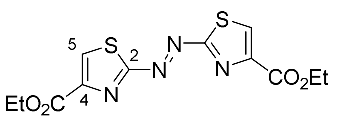

The hydrazine **4** (5.30 g, 15.5 mmol) was suspended in nitric acid (*w* = 65%, 6.0 mL), stirred for 5 min and left standing for 16 h (CAUTION: evolution of nitrous gases). After the addition of H_2O (240 mL) the product **2g** was filtered, thoroughly washed with H_2O, dried in a vacuum desiccator over $CaCl_2$ and obtained as ochre-colored, amorphous solid (4.73 g, 13.8 mmol, 69% o. 2 steps); mp > 245 °C (dec.) (lit.: 244–245 °C[3]). – ^{1}H-NMR (400 MHz, $CDCl_3$): δ = 1.45 (t, ^3J = 7 Hz, 6 H, 4-$CO_2CH_2\underline{CH_3}$), 4.50 (q, ^3J = 7 Hz, 4 H, 4-$CO_2\underline{CH_2}CH_3$), 8.40 (s, 2 H, 5-H). – ^{13}C-NMR (100 MHz, $CDCl_3$): δ = 14.4 (4-$CO_2CH_2\underline{CH_3}$), 62.1 (4-$CO_2\underline{CH_2}CH_3$), 131.2 (C5), 148.7 (C4), 160.7 (4-$\underline{C}O_2CH_2CH_3$), 173.8 (C2). – HRMS (ESI$^+$): *m*/*z* calcd for $C_{12}H_{13}N_4O_4S_2$ $[M + H]^+$ 341.0373, found 341.0374.

*1.2.2 Synthesis of the azothiazole derivatives **7a**–**f** and **8a**–**f***

Ethyl (*E*)-5-(butylamino)-2-[(4-(ethoxycarbonyl)thiazol-2-yl)diazenyl]thiazole-4-carboxylate (**7a**) (cf. Table 1, Entry 2)

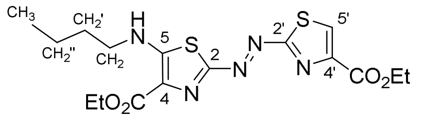

To a suspension of azothiazole **2g** (170 mg, 500 µmol) in $CHCl_3$ (6 mL) was added DABCO (112 mg, 1.00 mmol), which resulted in a color change from ochre to green. *n*-Butylamine (**5a**) (54.9 mg, 750 µmol, 74.1 µL) in $CHCl_3$ (4 mL) was added, which resulted in a color

change to red. The reaction mixture was stirred for 24 h at r.t. (TLC control). The reaction mixture was diluted with $CHCl_3$ (100 mL). The organic layer was washed with aq. HCl (c = 2 M, 2 x 25 mL), $NaHCO_3$ solution (sat., 50 mL) and NaCl solution (half saturated, 50 mL) dried with Na_2SO_4 and filtered from the drying agent. The solvent was removed under reduced pressure. The product was isolated by flash column chromatography (SiO_2; cyclohexane/EtOAc 7/3). The red fractions (R_f = 0.32) were combined and **7a** was obtained as golden, amorphous solid (44.0 mg, 107 µmol, 21%); mp 188–191 °C (dec.) – ^{1}H-NMR (500 MHz, $CDCl_3$): δ = 1.00 (t, ^3J = 8 Hz, 3 H, CH_3), 1.44 (t, ^3J = 7 Hz, 3 H, 4-$CO_2CH_2\underline{CH_3}$), 1.44 (t, ^3J = 7 Hz, 3 H, 4'-$CO_2CH_2\underline{CH_3}$), 1.44–1.52 (m, 2 H, CH_2''), 1.73–1.79 (m, 2 H, CH_2'), 3.37–3.41 (m, 2 H, CH_2), 4.44 (q, ^3J = 7 Hz, 2 H, 4-$CO_2\underline{CH_2}CH_3$), 4.46 (q, ^3J = 7 Hz, 2 H, 4'-$CO_2\underline{CH_2}CH_3$), 8.20 (s, 1 H, 5'-H), 8.46 (t, ^3J = 6 Hz, 1 H, NH). – ^{13}C-NMR (125 MHz, $CDCl_3$): δ = 13.6 (CH_3), 14.3 (4'-$CO_2CH_2\underline{CH_3}$), 14.5 (4-$CO_2CH_2\underline{CH_3}$), 20.0 (CH_2''), 30.5 (CH_2'), 49.5 (CH_2), 61.3 (4-$CO_2\underline{CH_2}CH_3$), 61.7 (4'-$CO_2\underline{CH_2}CH_3$), 125.0 (C4), 129.0 (C5'), 147.5 (C4'), 154.0 (C2), 161.2 (4'-$\underline{C}O_2CH_2CH_3$), 165.0 (4-$\underline{C}O_2CH_2CH_3$), 166.6 (C5), 175.3 (C2'). – MS (ESI$^+$): m/z = 412 (18) $[M + H]^+$, 434 (76) $[M + Na]^+$, 845 (100) $[2M + Na]^+$, 1256 (27) $[3M + Na]^+$. MS (ESI$^-$): m/z = 410 (100) $[M - H]^-$. – HRMS (ESI$^+$): m/z calcd for $C_{16}H_{22}N_5O_4S_2$ $[M + H]^+$ 412.1108, found 412.1106.

Ethyl (E)-5-((3-(dimethylamino)propyl)amino)-2-((4-(ethoxycarbonyl)thiazol-2-yl)diazenyl)thiazole-4-carboxylate (**7b**) (cf. Table 1, Entry 5)

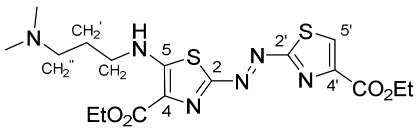

To a suspension of azothiazole **2g** (170 mg, 500 µmol) in $CHCl_3$ (6 mL) was added N,N'-dimethyl-1,3-propanediamine (**5b**) (76.6 mg, 750 µmol, 94.3 µL) in $CHCl_3$ (4 mL), which resulted in a color change to red. The reaction mixture was stirred for 2 h at r.t. (TLC control). The reaction mixture was diluted with $CHCl_3$ (150 mL). The organic layer was washed with water (40 mL) and NaCl solution (half saturated, 40 mL), dried with Na_2SO_4 and filtered from the drying agent. The solvent was removed under reduced pressure. The product was isolated by flash column chromatography (SiO_2; eluent: $CHCl_3$/MeOH 9/1). The red fractions (R_f = 0.24) were combined and **7b** was obtained as purple, amorphous solid (95.0 mg, 250 µmol, 50%); mp 164–167 °C (dec.) – ^{1}H-NMR (400 MHz, $CDCl_3$): δ = 1.43 (t, ^3J = 7 Hz, 3 H, 4-$CO_2CH_2\underline{CH_3}$), 1.43 (t, ^3J = 7 Hz, 3 H, 4'-$CO_2CH_2\underline{CH_3}$), 1.90 (tt, ^3J = 6 Hz, ^3J = 6 Hz, 2 H, CH_2'), 2.29 (s, 6 H, NMe_2), 2.50 (t, ^3J = 6 Hz, 2 H, CH_2''), 3.47 (t, ^3J = 6 Hz, 2 H, CH_2), 4.43 (q, ^3J = 7 Hz, 2 H, 4-$CO_2\underline{CH_2}CH_3$), 4.46 (q, ^3J = 7 Hz, 2 H, 4'-$CO_2\underline{CH_2}CH_3$), 8.17 (s, 1 H, 5'-H), 9.76 (br. s, 1 H, 5-NH). – ^{13}C-NMR (100 MHz, $CDCl_3$): δ = 14.4 (4'-$CO_2CH_2\underline{CH_3}$), 14.5 (4-$CO_2CH_2\underline{CH_3}$), 25.2 (CH_2'), 45.2 (NMe_2), 49.9 (CH_2), 57.7 (CH_2''), 61.0 (4-$CO_2\underline{CH_2}CH_3$), 61.6 (4'-$CO_2\underline{CH_2}CH_3$), 125.6 (C4), 128.7 (C5'), 147.3 (C4'), 153.5 (C2), 161.2 (4'-

$\underline{C}O_2CH_2CH_3$), 164.2 (4-$\underline{C}O_2CH_2CH_3$), 166.4 (C5), 175.6 (C2'). – HRMS (ESI$^+$): *m*/*z* calcd for $C_{17}H_{25}N_6O_4S_2$ [M + H]$^+$ 441.1373, found 441.1374.

Ethyl (*E*)-5-((3-((*tert*-butoxycarbonyl)amino)propyl)amino)-2-((4-(ethoxycarbonyl)thiazol-2-yl)diazenyl)thiazole-4-carboxylate (**7c**) (cf. Table 1, Entry 7)

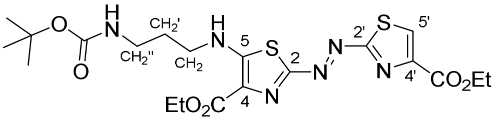

N-Boc-1,3-propanediamine (**5c**) was prepared according to a published procedure[4] from propane-1,3-diamine (22.2 g, 300 mmol, 25.3 mL) and di-*tert*-butyl dicarbonate (6.55 g, 30.0 mmol) and obtained as colorless oil that solidified on standing (4.74 g, 27.2 mmol, 91%). The ^{1}H-NMR spectroscopic data matched the reported ones.[4]

To a suspension of azothiazole **2g** (85.1 mg, 250 µmol) in $CHCl_3$ (15 mL) was added *N*-Boc-1,3-propanediamine (**5c**) (174 mg, 1.00 mmol) in $CHCl_3$ (3 mL), which resulted in a color change to red. The reaction mixture was stirred for 120 d at r.t. (TLC control). The reaction mixture was diluted with $CHCl_3$ (30 mL). The organic layer was washed with aq. HCl (*c* = 1 M, 12 mL), H_2O (20 mL) and NaCl solution (half saturated, 20 mL), dried with Na_2SO_4 and filtered from the drying agent. The solvent was removed under reduced pressure. The product was isolated by flash column chromatography (SiO_2; cyclohexane/EtOAc 1/1). The red fractions (R_f = 0.38) were combined and **7c** was obtained as red, amorphous solid (21.0 mg, 41.0 µmol, 16%); mp 169–174 °C (dec.) – ^{1}H-NMR (600 MHz, $CDCl_3$): δ = 1.41 (t, ^3J = 7 Hz, 3 H, 4-$CO_2CH_2\underline{CH_3}$), 1.41 (t, ^3J = 7 Hz, 3 H, 4'-$CO_2CH_2\underline{CH_3}$), 1.43 [s, 9 H, $CO_2C\underline{(CH_3)_3}$], 1.91 (tt, ^3J = 6 Hz, ^3J = 6 Hz, 2 H, CH_2'), 3.26 [dt, ^3J = 6 Hz, ^3J = 6 Hz, 2 H, CH_2''), 3.44 [dt, ^3J = 6 Hz, ^3J = 6 Hz, 2 H, CH_2), 4.42 (q, ^3J = 7 Hz, 2 H, 4-$CO_2\underline{CH_2}CH_3$), 4.43 (q, ^3J = 7 Hz, 2 H, 4'-$CO_2\underline{CH_2}CH_3$), 4.81 [br. s, 1 H, $\underline{NH}CO_2C(CH_3)_3$], 8.18 (s, 1 H, 5'-H), 8.68 (br. s, 1 H, 5-NH). – ^{13}C-NMR (150 MHz, $CDCl_3$): δ = 14.3 (4'-$CO_2CH_2\underline{CH_3}$), 14.4 (4-$CO_2CH_2\underline{CH_3}$), 28.3 [$CO_2C\underline{(CH_3)_3}$], 29.1 ($CH_2$'), 37.4 ($CH_2$''), 47.1 ($CH_2$), 61.2 (4-$CO_2\underline{CH_2}CH_3$), 61.6 (4'-$CO_2\underline{CH_2}CH_3$), 79.7 [$CO_2\underline{C}(CH_3)_3$], 125.4 (C4), 128.9 (C5'), 147.4 (C4'), 153.8 (C2), 156.4 [$\underline{C}O_2C(CH_3)_3$], 161.1 (4'-$\underline{C}O_2CH_2CH_3$), 164.5 (4-$\underline{C}O_2CH_2CH_3$), 166.2 (C5), 175.2 (C2'). – HRMS (ESI$^+$): *m*/*z* calcd for $C_{20}H_{29}N_6O_6S_2$ [M + H]$^+$ 513.1585, found 513.1585.

Ethyl (*E*)-2-((4-(ethoxycarbonyl)thiazol-2-yl)diazenyl)-5-(*p*-tolylamino)thiazole-4-carboxylate (**7d**) (cf. Table 1, Entry 12)

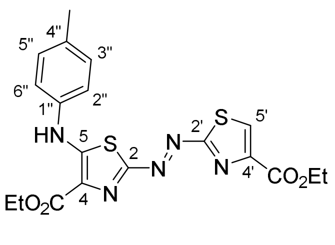

To a suspension of azothiazole **2g** (170 mg, 500 µmol) in MeCN (6 mL) was added DABCO (112 mg, 1.00 mmol), which resulted in a color change from ochre to green. *p*-Toluidine (**5d**)

(161 mg, 1.50 mmol) in MeCN (4 mL) was added, which resulted in a color change to red/purple. The reaction mixture was stirred for 7 d at r.t. (TLC control). The solvent was removed under reduced pressure. The residue was dissolved in $CHCl_3$ (200 mL). The organic layer was washed with aq. HCl (c = 2 M, 2 x 25 mL) and NaCl solution (half saturated, 50 mL), dried with Na_2SO_4 and filtered from the drying agent. The solvent was removed under reduced pressure. The product was isolated by flash column chromatography (SiO_2; cyclohexane/EtOAc 4/1). The red fractions (R_f = 0.31) were combined. The product **7d** was further purified by column chromatography (SiO_2; $CHCl_3$, R_f = 0.23), washed with *n*-hexane and obtained as red, amorphous solid (41 mg, 92 µmol, 18%); mp > 225 °C (dec.) – ^{1}H-NMR (500 MHz, $CDCl_3$): δ = 1.43 (t, ^3J = 7 Hz, 3 H, 4'-$CO_2CH_2\underline{CH_3}$), 1.48 (t, ^3J = 7 Hz, 3 H, 4'-$CO_2CH_2\underline{CH_3}$), 2.39 (s, 3 H, 4''-CH_3), 4.46 (q, ^3J = 7 Hz, 2 H, 4'-$CO_2\underline{CH_2}CH_3$), 4.51 (q, ^3J = 7 Hz, 2 H, 4-$CO_2\underline{CH_2}CH_3$), 7.23–7.28 (m, 4 H, 2''-H, 3''-H, 5''-H, 6''-H), 8.23 (s, 1 H, 5'-H), 10.54 (s, 1 H, 5-NH). – ^{13}C-NMR (125 MHz, $CDCl_3$): δ = 14.3 (4'-$CO_2CH_2\underline{CH_3}$), 14.5 (4-$CO_2CH_2\underline{CH_3}$), 21.0 (4''-CH_3), 61.7 (4'-$CO_2\underline{CH_2}CH_3$), 61.7 (4-$CO_2\underline{CH_2}CH_3$), 119.8 (C2'', C6''), 126.9 (C4), 129.3 (C5'), 130.5 (C3'', C5''), 136.3 (C1''), 136.3 (C4''), 147.8 (C4'), 154.7 (C2), 160.2 (C5), 161.1 (4'-$\underline{C}O_2CH_2CH_3$), 165.2 (4-$\underline{C}O_2CH_2CH_3$), 174.9 (C2'). – HRMS (ESI$^+$): *m*/*z* calcd for $C_{19}H_{20}N_5O_4S_2$ [M + H]$^+$ 446.0951, found 446.0953.

Diethyl 2,2'-(diazene-1,2-diyl)-(*E*)-bis(5-(butylamino)thiazole-4-carboxylate) (**8a**) (cf. Table 1, Entry 3)

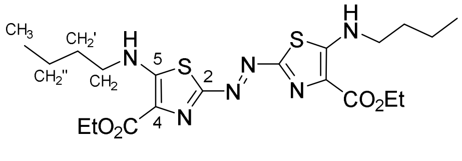

To a suspension of azothiazole **2g** (170 mg, 500 µmol) in THF (40 mL) was added *n*-butylamine (**5a**) (110 mg, 1.50 mmol, 148 µL), which resulted in an immediate color change to purple. The reaction mixture was stirred for 12 d at r.t., whereas additional amine **5a** (50 µL each) was added after 8 and 9 days of stirring (TLC control on SiO_2; cyclohexane/EtOAc 4/1; R_f = 0.23 for bis-substituted purple product **8a**, R_f = 0.13 for mono-substituted red product **7a**). H_2O (50 mL) was added and the reaction mixture was extracted with $CHCl_3$ (2 x 100 mL). The combined organic layers were washed with NaCl solution (half-saturated, 50 mL), dried with Na_2SO_4 and filtered from the drying agent. The solvent was removed under reduced pressure. The product was isolated by column chromatography (SiO_2; cyclohexane/EtOAc 4/1). The purple fractions (R_f = 0.23) were combined and **8a** was obtained as golden, amorphous solid (68.0 mg, 141 µmol, 28%). Further crystallization from EtOAc at –20 °C gave **8a** in the form of fine, golden needles; mp = 214–216 °C (dec.). –^{1}H-NMR (400 MHz, $CDCl_3$): δ = 0.98 (t, ^3J = 7 Hz, 6 H, CH_3), 1.43 (t, ^3J = 7 Hz, 6 H, 4-$CO_2CH_2\underline{CH_3}$), 1.41–1.51 (m, 4 H, CH_2''), 1.69–1.76 (m, 4 H, CH_2'), 3.34 (dt, ^3J = 7 Hz, ^3J = 6 Hz, 4 H, CH_2), 4.41 (q, ^3J = 7 Hz, 4 H, 4-$CO_2\underline{CH_2}CH_3$), 8.15 (t, ^3J = 6 Hz, 2 H, 5-NH). – ^{13}C-NMR (100 MHz, $CDCl_3$): δ = 13.6 (CH_3), 14.6 (4-$CO_2CH_2\underline{CH_3}$), 20.0 (CH_2''), 30.7 (CH_2'), 49.2

(CH_2), 60.8 (4-$CO_2\underline{CH_2}CH_3$), 122.4 (C4), 155.4 (C2), 165.2 (4-$\underline{C}O_2CH_2CH_3$), 165.2 (C5). – MS (ESI$^+$): *m/z* = 483 (40) [M + H]$^+$, 505 (100) [M + Na]$^+$, 987 (87) [2M + Na]$^+$, 1469 (23) [3M + Na]$^+$. – MS (ESI$^-$): *m/z* = 481 (100) [M – H]$^-$. – HRMS (ESI$^+$): *m*/*z* calcd for $C_{20}H_{31}N_6O_4S_2$ [M+H]$^+$ 483.1843, found 483.1847.

Diethyl 2,2'-(diazene-1,2-diyl)-(*E*)-bis(5-((3-(dimethyl-amino)propyl)amino)thiazole-4-carboxylate) (**8b**) (cf. Table 1, Entry 6)

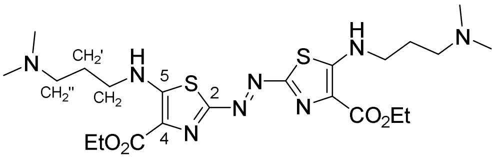

To a suspension of azothiazole **2g** (102 mg, 300 µmol) in THF (24 mL) was added *N,N'*-dimethyl-1,3-propanediamine (**5b**) (91.7 mg, 900 µmol, 113 µL), which resulted in an immediate color change to purple. The reaction mixture was stirred for 9 d at r.t., whereas additional amine **5b** (40 µL) was added after 1 day of stirring (TLC control on SiO_2: $CHCl_3$/MeOH 9/1; R_f = 0.07 for bis-substituted purple product **8b**, R_f = 0.24 for mono-substituted red product **7b**). A solution of $NaHCO_3$ (sat.; 50 mL) was added and the reaction mixture was extracted with $CHCl_3$ (3 x 100 mL). The solvent was removed under reduced pressure. The product **8b** was isolated by crystallization from MeOH (50 °C → –20 °C) as dark green, amorphous solid (61.0 mg, 113 µmol, 38%); mp = 182–185 °C (dec.). – ^{1}H-NMR (400 MHz, $CDCl_3$): δ = 1.41 (t, ^3J = 7 Hz, 6 H, 4-$CO_2CH_2\underline{CH_3}$), 1.86 (tt, ^3J = 6 Hz, ^3J = 6 Hz, 4 H, CH_2'), 2.25 (s, 12 H, NMe_2), 2.43 (t, ^3J = 6 Hz, 4 H, CH_2''), 3.40 (dt, ^3J = 6 Hz, ^3J = 5 Hz, 4 H, CH_2), 4.40 (q, ^3J = 7 Hz, 4 H, 4-$CO_2\underline{CH_2}CH_3$), 8.85 (br. s, 2 H, 5-NH). – ^{13}C-NMR (100 MHz, $CDCl_3$): δ = 14.6 (4-$CO_2CH_2\underline{CH_3}$), 26.0 (CH_2'), 45.3 (NMe_2), 48.8 (CH_2), 57.3 (CH_2''), 60.6 (4-$CO_2\underline{CH_2}CH_3$), 122.5 (C4), 155.3 (C2), 164.7 (4-$\underline{C}O_2CH_2CH_3$), 165.0 (C5). – MS (ESI$^+$): *m/z* = 541 (100) [M + H]$^+$, 563 (80) [M + Na]$^+$, 1081 (45) [2M + H]$^+$, 1103 (30) [2M + Na]$^+$. – HRMS (ESI$^+$): *m*/*z* calcd for $C_{22}H_{37}N_8O_4S_2$ [M + H]$^+$ 541.2374, found 541.2374.

Diethyl 2,2'-(diazene-1,2-diyl)-(*E*)-bis(5-((3-((tert-butoxy-carbonyl)amino)propyl)amino)thiazole-4-carboxylate) (**8c**) (cf. Table 1, Entry 11)

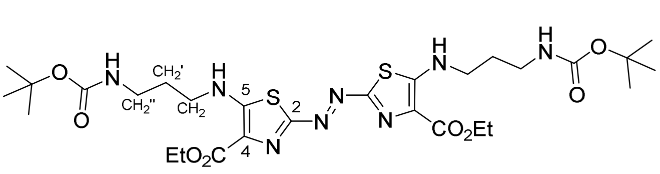

To a suspension of azothiazole **2g** (170 mg, 500 µmol) in MeCN (6 mL) was added DABCO (112 mg, 1.00 mmol), which resulted in a color change from ochre to green. *N*-Boc-1,3-propanediamine[4] (**5c**) (261 mg, 1.50 mmol) in MeCN (4 mL) was added, which resulted in a color change to red/purple. The reaction mixture was stirred for 16 h at r.t. (TLC control on SiO_2; cyclohexane/EtOAc 1/1; R_f = 0.29 for bis-substituted purple product **8c**, R_f = 0.38 for

mono-substituted red product **7c**). The solvent was removed under reduced pressure. The residue was dissolved in $CHCl_3$ (200 mL). The organic layer was washed with aq. HCl (c = 2 M, 2 x 25 mL), $NaHCO_3$ solution (sat., 50 mL) and NaCl solution (half saturated, 50 mL), dried with Na_2SO_4 and filtered from the drying agent. The solvent was removed under reduced pressure. The crude product was isolated by flash column chromatography (SiO_2; cyclohexane/EtOAc 1/1). The purple fractions (R_f = 0.29) were combined and **8c** was obtained as purple, amorphous solid (120 mg, 175 µmol, 35%); mp = 210–212 °C (dec.) – ^{1}H-NMR (400 MHz, $CDCl_3$): δ = 1.42 (t, ^3J = 7 Hz, 6 H, 4-$CO_2CH_2\underline{CH_3}$), 1.46 [s, 18 H, $CO_2C\underline{(CH_3)_3}$], 1.90 (tt, ^3J = 6 Hz, ^3J = 6 Hz, 4 H, CH_2'), 3.26 (dt, ^3J = 6 Hz, ^3J = 6 Hz, 4 H, CH_2''), 3.40 (dt, ^3J = 6 Hz, ^3J = 6 Hz, 4 H, CH_2), 4.41 (q, ^3J = 7 Hz, 4 H, 4-$CO_2\underline{CH_2}CH_3$), 4.67 [br. s, 2 H, $\underline{NH}CO_2C(CH_3)_3$], 8.30 (br. s, 2 H, 5-NH). – ^{13}C-NMR (100 MHz, $CDCl_3$): δ = 14.5 (4-$CO_2CH_2\underline{CH_3}$), 28.4 [$CO_2C\underline{(CH_3)_3}$], 29.4 ($CH_2$'), 37.7 ($CH_2$''), 46.8 ($CH_2$), 60.9 (4-$CO_2\underline{CH_2}CH_3$), 79.7 [$CO_2\underline{C}(CH_3)_3$], 122.8 (C4), 155.4 (C2), 156.2 [$\underline{C}O_2C(CH_3)_3$], 164.9 (4-$\underline{C}O_2CH_2CH_3$), 164.9 (C5). – HRMS ($ESI^+$): m/z calcd for $C_{28}H_{45}N_8O_8S_2$ $[M + H]^+$ 685.2796, found 685.2777.

Diethyl 2,2'-(diazene-1,2-diyl)-(*E*)-bis(5-(*p*-tolylamino)thiazole-4-carboxylate) (**8d**) (cf. Table 1, Entry 12)

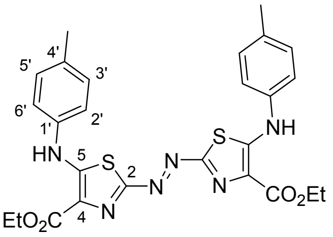

The product **8d** was isolated as side product during the synthesis of **7d** by flash column chromatography (SiO_2; cyclohexane/EtOAc 4/1). The blue fractions (R_f = 0.44) were combined. The product **8d** was further purified by column chromatography (SiO_2; $CHCl_3$, R_f = 0.42), washed with *n*-hexane and obtained as golden, amorphous solid (7.0 mg, 13 µmol, 3%). – ^{1}H-NMR (500 MHz, $CDCl_3$): δ = 1.47 (t, ^3J = 7 Hz, 6 H, 4-$CO_2CH_2\underline{CH_3}$), 2.38 (s, 6 H, 4'-CH_3), 4.48 (q, ^3J = 7 Hz, 4 H, 4-$CO_2\underline{CH_2}CH_3$), 7.21–7.25 (m, 8 H, 2'-H, 3'-H, 5'-H, 6'-H), 10.35 (s, 2 H, 5-NH). – ^{13}C-NMR (125 MHz, $CDCl_3$): δ = 14.5 (4-$CO_2CH_2\underline{CH_3}$), 20.9 (4'-CH_3), 61.3 (4-$CO_2\underline{CH_2}CH_3$), 119.4 (C2', C6'), 125.2 (C4), 130.4 (C3', C5'), 135.4 (C4'), 136.8 (C1'), 155.9 (C2), 158.7 (C5), 165.4 (4-$\underline{C}O_2CH_2CH_3$). – HRMS (ESI^+): m/z calcd for $C_{26}H_{27}N_6O_4S_2$ $[M + H]^+$ 551.1530, found 551.1533.

Diethyl 2,2'-(diazene-1,2-diyl)-(*E*)-bis(5-((4-(dimethylamino)phenyl)amino)thiazole-4-carboxylate) (**8e**) (cf. Table 1, Entry 13)

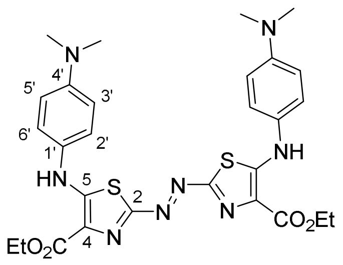

N,*N*-Dimethyl-*p*-phenylenediamine (**5e**) was distilled prior to use (0.014 mbar, 70–72 °C). The obtained yellow oil was stored under an argon atmosphere at 4 °C.
To a suspension of azothiazole **2g** (170 mg, 500 µmol) in MeCN (6 mL) was added DABCO (112 mg, 1.00 mmol), which resulted in a color change from ochre to green. *N*,*N*-Dimethyl-*p*-phenylenediamine (**5e**) (272 mg, 2.00 mmol, 250 µL) in MeCN (4 mL) was added, which resulted in a color change to red/purple. The reaction mixture was stirred for 7 d at r.t, whereas additional amine **5e** (~200 µL) was added after 2 days of stirring. The solvent was removed under reduced pressure. The residue was dissolved in $CHCl_3$ (200 mL). The organic layer was washed with water (50 mL) and NaCl solution (half saturated, 50 mL), dried with Na_2SO_4 and filtered from the drying agent. The solvent was removed under reduced pressure. The product was isolated by two-fold flash column chromatography [firstly: SiO_2; $CHCl_3$ (R_f = 0.19), then $CHCl_3$/MeOH 200/1 and secondly: SiO_2; cyclohexane/EtOAc 1/1 (R_f = 0), then $CHCl_3$/MeOH 98/2]. For further purification the blue fraction was subjected to column chromatography (Al_2O_3 neutral, activity grade I; $CHCl_3$/cyclohexane, R_f = 0.46). The product **8e** was washed with *n*-hexane and obtained as dark blue, amorphous solid (4.4 mg, 7.2 µmol, 1%). – ^{1}H-NMR (500 MHz, $CDCl_3$): δ = 1.46 (t, ^3J = 7 Hz, 6 H, 4-$CO_2CH_2\underline{CH_3}$), 3.00 (s, 12 H, 4'-NMe_2), 4.46 (q, ^3J = 7 Hz, 4 H, 4-$CO_2\underline{CH_2}CH_3$), 6.74 (d, ^3J = 9 Hz, 4 H, 3'-H, 5'-H), 7.21 (d, ^3J = 9 Hz, 4 H, 2'-H, 6'-H), 10.09 (s, 2 H, 5-NH). – ^{13}C-NMR (125 MHz, $CDCl_3$): δ = 14.6 (4-$CO_2CH_2\underline{CH_3}$), 40.6 (4'-NMe_2), 61.1 (4-$CO_2\underline{CH_2}CH_3$), 113.0 (C3', C5'), 121.6 (C2', C6'), 124.4 (C4), 128.8 (C1'), 148.7 (C4'), 155.4 (C2), 160.2 (C5), 165.5 (4-$\underline{C}O_2CH_2CH_3$). – HRMS (ESI$^+$): *m*/*z* calcd for $C_{28}H_{33}N_8O_4S_2$ $[M+H]^+$ 609.2061, found 609.2041.

*General procedure for the deprotection of the Boc-protected azothiazole derivatives **7c** and **8c** (GP1)*

To a solution of the Boc-protected azothiazole derivatives **7c** and **8c** in CH_2Cl_2 was added dropwise TFA at 0 °C. The solution was stirred for 2 h at 0 °C and for 2 h at r.t (TLC control). The solvent was removed under reduced pressure. In order to remove excess TFA the residue was redissolved in CH_2Cl_2 (20 mL) and the solvent was removed under reduced pressure. This process was repeated four times. The product was finally washed with CH_2Cl_2 (2 x 50 mL) and dried.

(*E*)-3-((4-(Ethoxycarbonyl)-2-((4-(ethoxycarbonyl)thiazol-2-yl)diazenyl)thiazol-5-yl)amino)propan-1-aminium trifluoroacetate (**7f**)

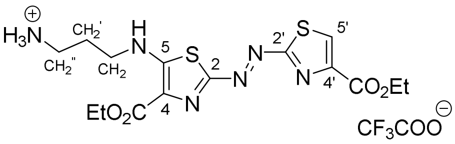

The azothiazole derivative **7c** (64.1 mg, 125 µmol) in CH_2Cl_2 (8 mL) was treated with TFA (1 mL) according to GP1. The product **7f** was obtained as red, amorphous solid (63.0 mg, 120 µmol, 96%); mp 180–183 °C (dec.). – R_f (SiO_2, $CHCl_3$/MeOH 9/1) = 0.18. – ^{1}H-NMR (500 MHz, DMSO-d_6): δ = 1.33 (t, ^3J = 7 Hz, 3 H, 4'-$CO_2CH_2\underline{CH_3}$), 1.34 (t, ^3J = 7 Hz, 3 H, 4-$CO_2CH_2\underline{CH_3}$), 1.96 (tt, ^3J = 7 Hz, ^3J = 7 Hz, 2 H, CH_2'), 2.86–2.92 (m, 2 H, CH_2''), 3.53 [dt, ^3J = 6 Hz, ^3J = 6 Hz, 2 H, CH_2), 4.33 (q, ^3J = 7 Hz, 2 H, 4'-$CO_2\underline{CH_2}CH_3$), 4.35 (q, ^3J = 7 Hz, 2 H, 4'-$CO_2\underline{CH_2}CH_3$), 7.79 (br. s, 3 H, NH_3^+), 8.58 (s, 1 H, 5'-H), 9.21 (t, ^3J = 6 Hz, 1 H, 5-NH). – ^{13}C-NMR (125 MHz, DMSO-d_6): δ = 14.1 (4'-$CO_2CH_2\underline{CH_3}$), 14.3 (4-$CO_2CH_2\underline{CH_3}$), 25.3 (CH_2'), 36.5 (CH_2''), 47.1 (CH_2), 60.6 (4-$CO_2\underline{CH_2}CH_3$), 61.1 (4'-$CO_2\underline{CH_2}CH_3$), 125.7 (C4), 130.5 (C5'), 146.4 (C4'), 152.2 (C2), 160.4 (4'-$\underline{C}O_2CH_2CH_3$), 163.1 (4-$\underline{C}O_2CH_2CH_3$), 166.2 (C5), 174.6 (C2'). – HRMS (ESI$^+$): *m*/*z* calcd for $C_{15}H_{21}N_6O_4S_2$ $[M]^+$ 413.1060, found 413.1056.

(*E*)-3,3'-((Diazene-1,2-diylbis(4-(ethoxycarbonyl)thiazole-2,5-diyl))bis(azanediyl))bis(propan-1-aminium) bistrifluoroacetate (**8f**)

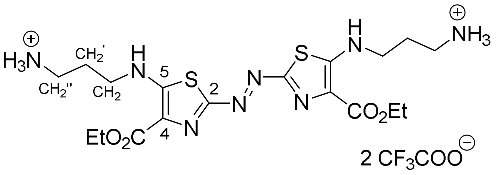

The azothiazole derivative **8d** (120 mg, 175 µmol) in CH_2Cl_2 (12 mL) was treated with TFA (1.5 mL) according to GP1. The product **8f** was obtained as purple, amorphous solid (120 mg, 163 µmol, 96%); mp 215–217°C (dec.). – ^{1}H-NMR (500 MHz, DMSO-d_6): δ = 1.32 (t, ^3J = 7 Hz, 6 H, 4-$CO_2CH_2\underline{CH_3}$), 1.93 (tt, ^3J = 7 Hz, ^3J = 7 Hz, 4 H, CH_2'), 2.84–2.90 (m, 4 H, CH_2''), 3.46 (dt, ^3J = 6 Hz, ^3J = 6 Hz, 4 H, CH_2), 4.30 (q, ^3J = 7 Hz, 4 H, 4-$CO_2\underline{CH_2}CH_3$), 7.81 (br. s, 6 H, NH_3^+), 8.73 (t, ^3J = 6 Hz, 2 H, 5-NH). – ^{13}C-NMR (125 MHz, DMSO-d_6): δ = 14.3 (4-$CO_2CH_2\underline{CH_3}$), 25.6 (CH_2'), 36.5 (CH_2''), 46.5 (CH_2), 60.1 (4-$CO_2\underline{CH_2}CH_3$), 122.3 (C4), 153.6 (C2), 163.5 (4-$\underline{C}O_2CH_2CH_3$), 164.3 (C5). – MS (ESI$^+$): *m*/*z* = 485 (90) $[M-H]^+$, 507 (100) $[M-2H+Na]^+$, 991 (87) $[2M-4H+Na]^+$, 1475 (32) $[3M-6H+Na]^+$. – HRMS (ESI$^+$): *m*/*z* calcd for $C_{18}H_{29}N_8O_4S_2$ $[M–H]^+$ 485.1748, found 485.1746.

2 Additional spectroscopic data

2.1 Absorption and emission properties

Table S1. Absorption properties of Azothiazole Derivatives **7f** and **8f.**

	**7f**		**8f**	
Solvent[a]	λ_{abs}[b]	lg ε[c]	λ_{abs}[b]	lg ε[c]
H_2O	-[d]	-[d]	579	4.58
MeOH	531	4.38	577	4.65
DMSO	587	4.72	590	4.67

[a]Solvents arranged in order of decreasing E_T^{30} values. [b]Long-wavelength absorption maximum in nm; c = 10 µM. [c]Molar extinction coefficient in cm^{-1} M^{-1}. [d]Not (fully) soluble.

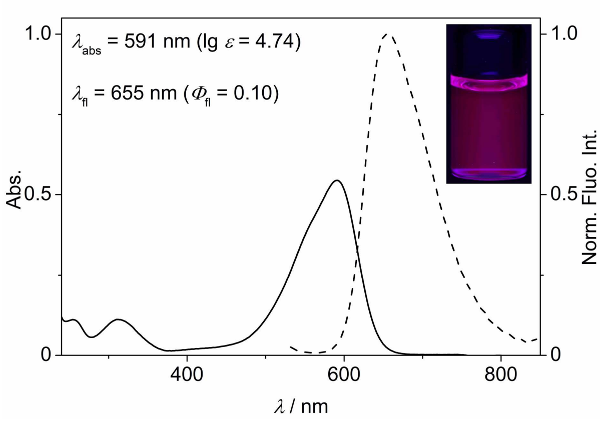

Figure S1. Absorption (solid line), normalized emission spectrum (λ_{ex} = 515 nm, dashed line) and fluorescence color (λ_{ex} = 366 nm) of derivative **8b** in glycerol (c = 10 µM with 1% DMSO).

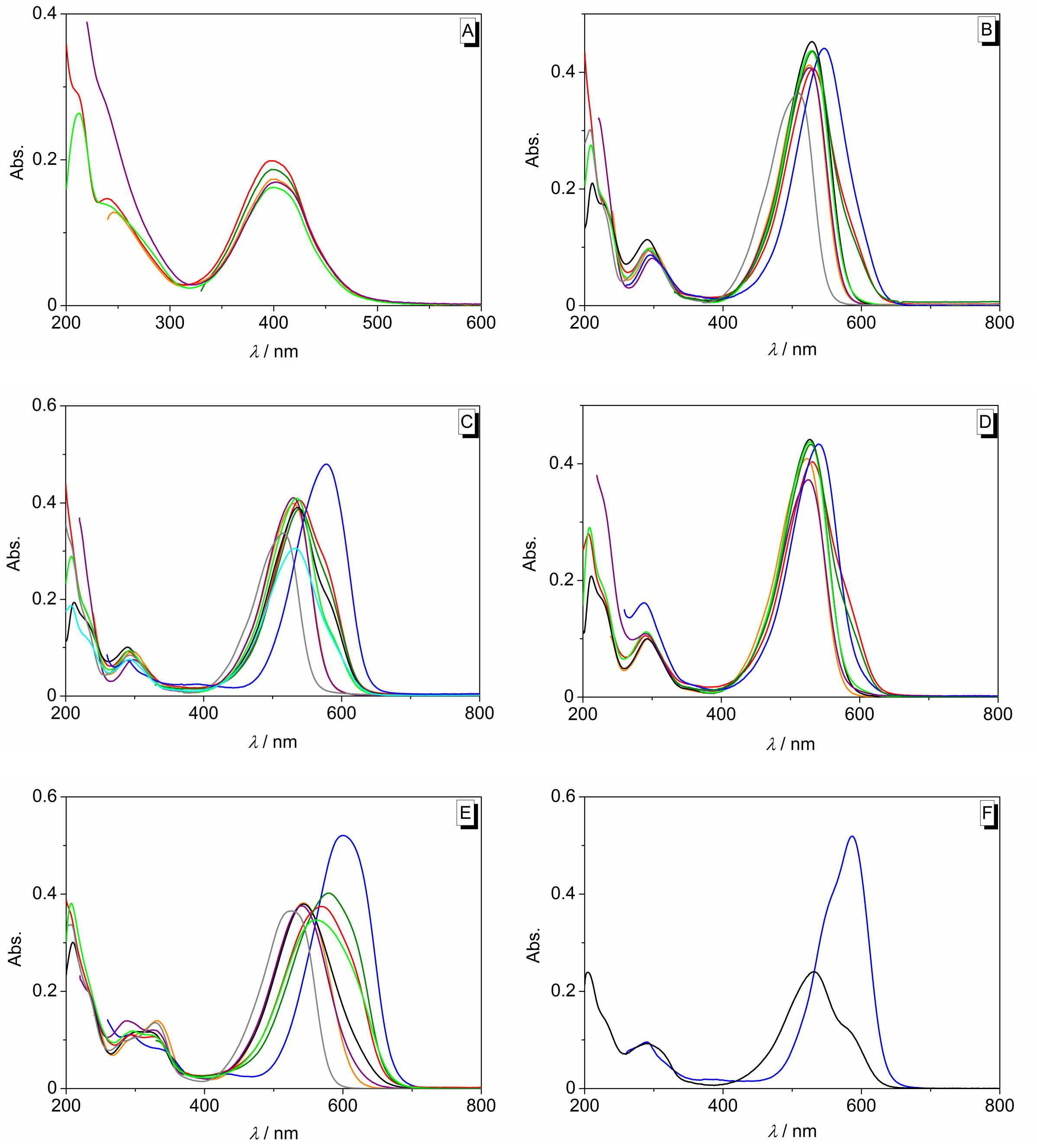

Figure S2. Absorption spectra of derivatives **2g** (A), **7a** (B), **7b** (C), **7c** (D), **7d** (E), **7f** (F); *c* = 10 µM; solvents: H_2O (cyan), MeOH (black), EtOH (green), MeCN (red), DMSO (blue), acetone (olive), $CHCl_3$ (orange), THF (purple), *n*-hexane (gray).

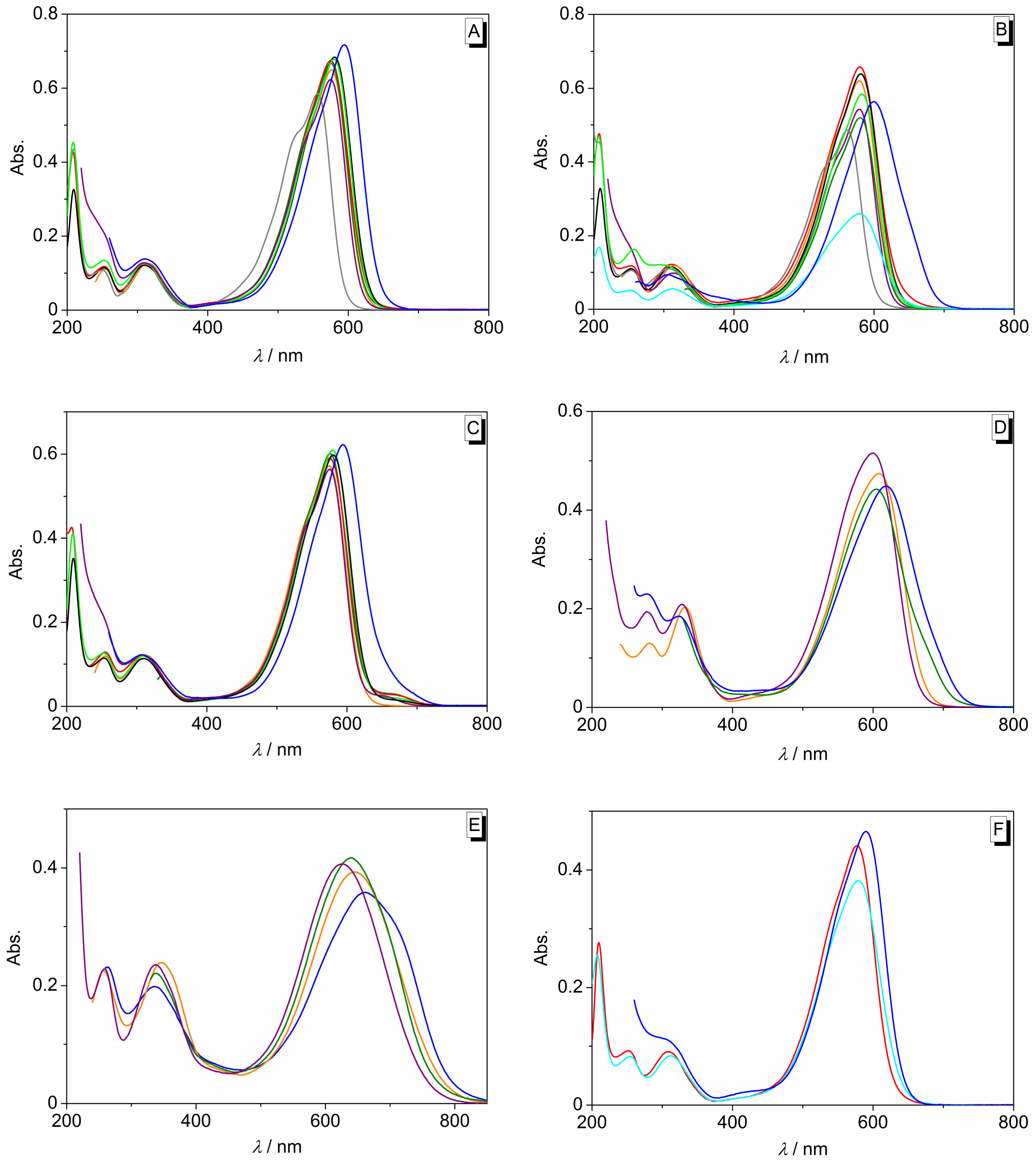

Figure S3. Absorption spectra of derivatives **8a** (A), **8b** (B), **8c** (C), **8d** (D), **8e** (E), **8f** (F); *c* = 10 μM; solvents: H_2O (cyan), MeOH (black), EtOH (green), MeCN (red), DMSO (blue), acetone (olive), $CHCl_3$ (orange), THF (purple), *n*-hexane (gray).

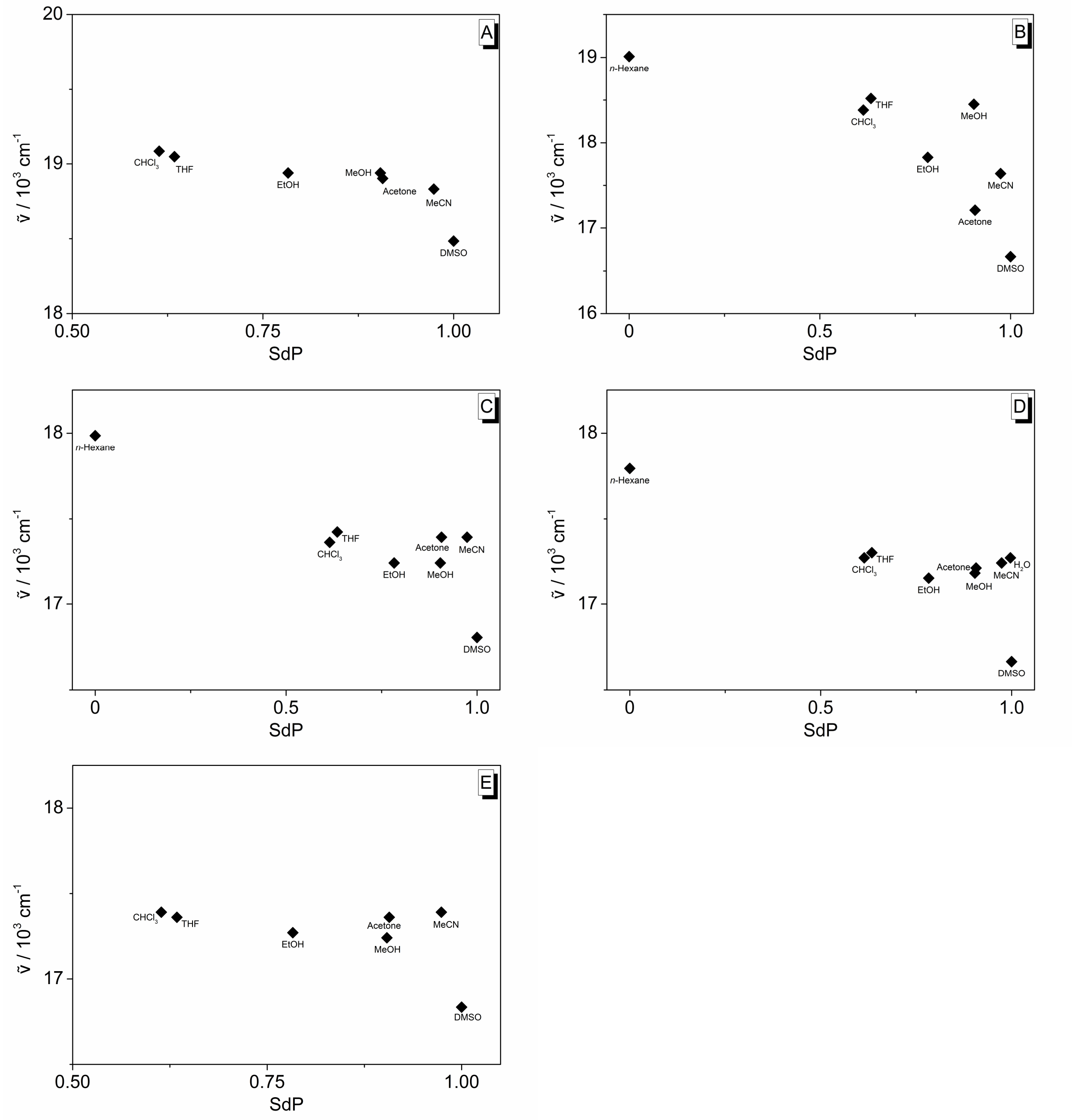

Figure S4. Plot of the absorption maximum of **7c** (A), **7d** (B) and **8a** (C), **8b** (D) and **8c** (E) in the respective solvents versus the dipolarity SDP.[5]

2.2 DNA-binding properties

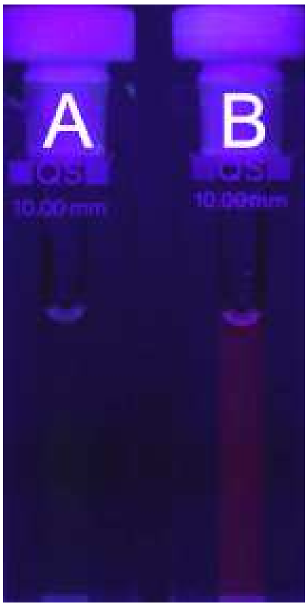

Figure S5. Fluorescence colors of **8b** (c_L = 10 µM) in the absence (A) and in the presence (B) of ct DNA (c_{DNA} = 300 µM); λ_{ex} = 366 nm. The contrast and brightness were enhanced by 30% without changing the true colors.

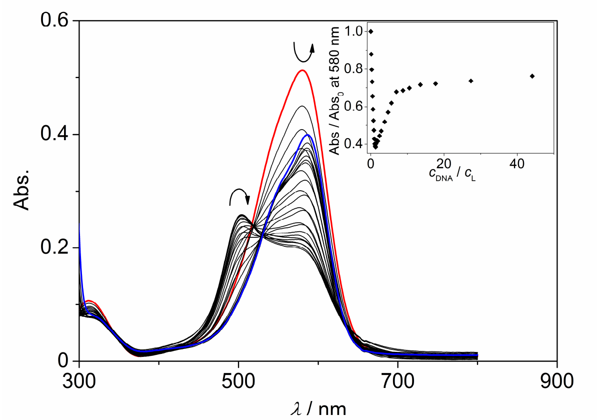

Figure S6. Spectrophotometric titration of **8f** with ct DNA (c_L = 10 µM, c_{DNA} = 2.17 mM; c_{DNA} in base pairs) in BPE buffer (c_{Na+}= 16 mM, pH 7.0; with 5% v/v DMSO). Red: Spectrum of the pure ligand solution; blue: spectrum at the end of the titration. The arrows indicate the changes of absorption upon addition of DNA. Inset: Plot of Abs. / Abs.$_0$ versus c_{DNA} / c_L.

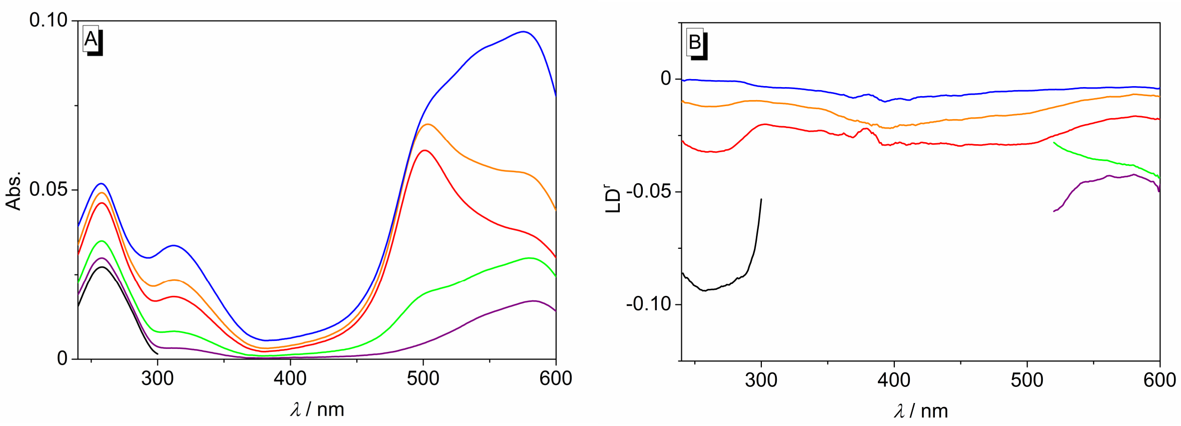

Figure S7. Absorption spectra (d = 1 mm) (A) and reduced LD spectra (LD^r = LD / Abs_{iso}) (B) of ct DNA (c = 20 µM) in the absence and presence of **8b** at LDR = 0 (black), 0.20 (purple), 0.50 (green), 1.00 (red), 1.50 (orange), 2.00 (blue) in BPE buffer (c_{Na+}= 16 mM, pH 7.0; with 5% v/v DMSO) (cf. Figure 4 B).

3 ^{1}H- and ^{13}C-NMR spectra

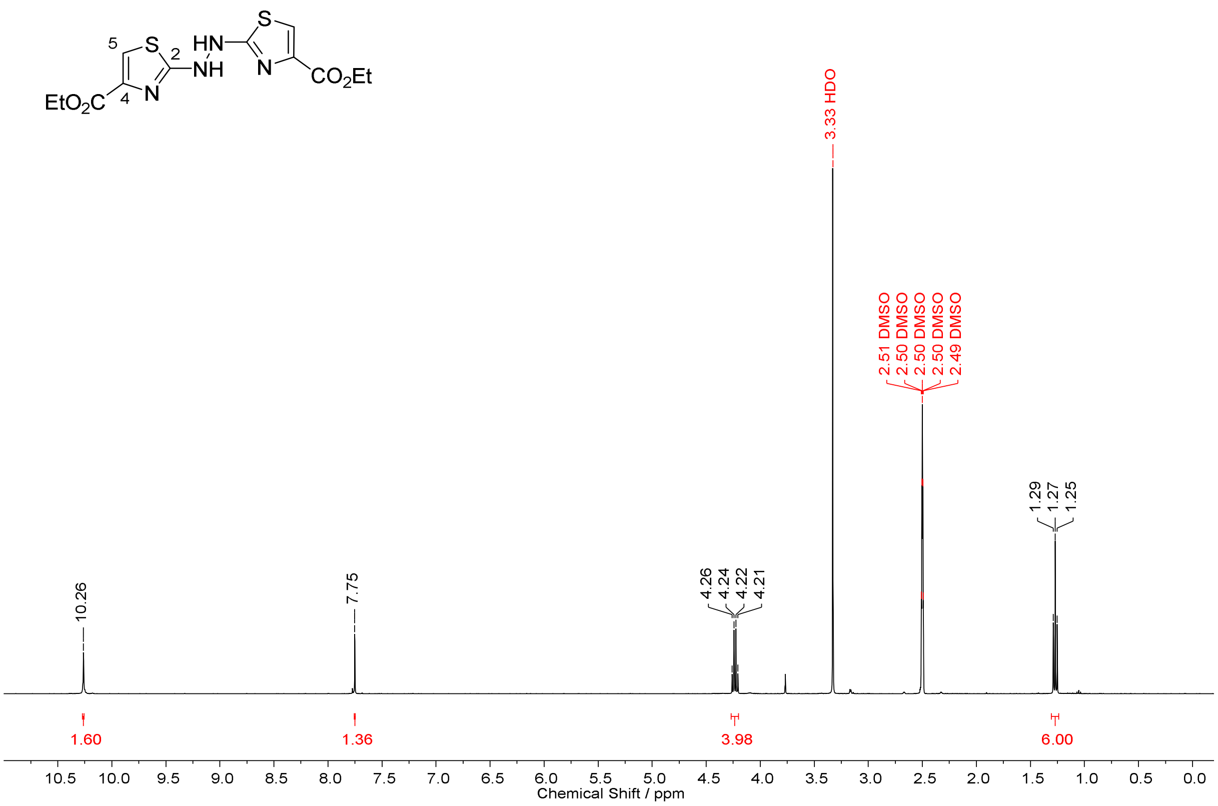

Figure S8. ^{1}H-NMR spectrum (400 MHz) of derivative **4** in DMSO-d_6.

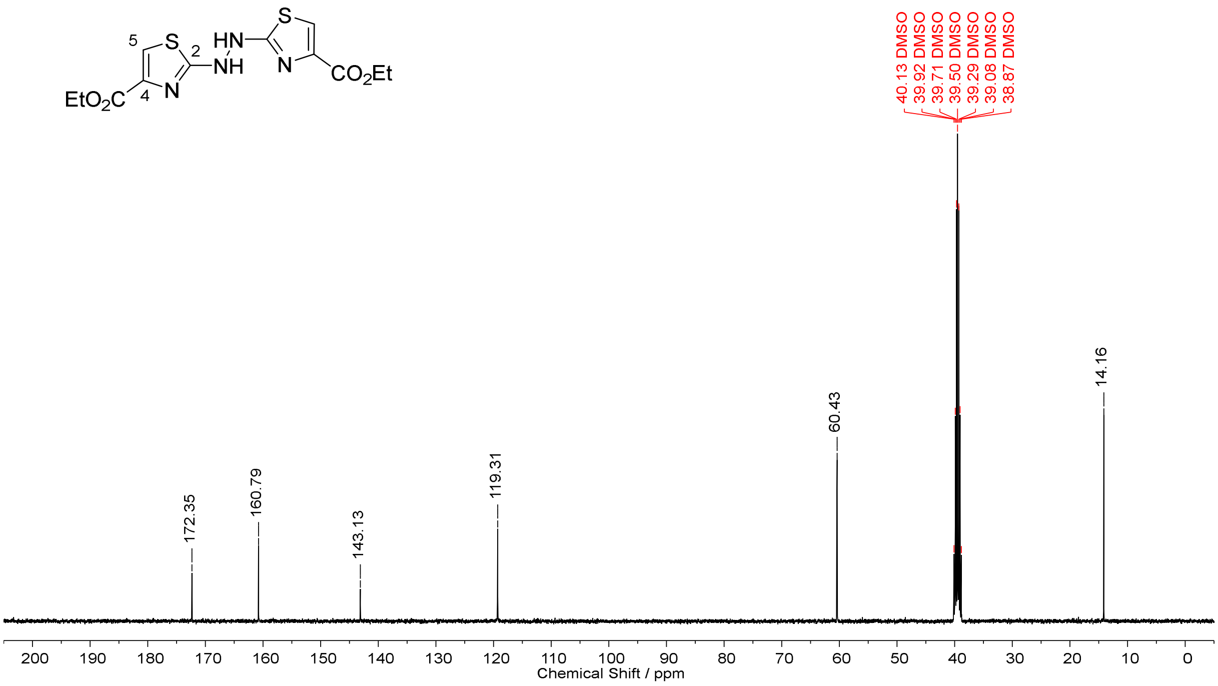

Figure S9. ^{13}C-NMR spectrum (100 MHz) of derivative **4** in DMSO-d_6.

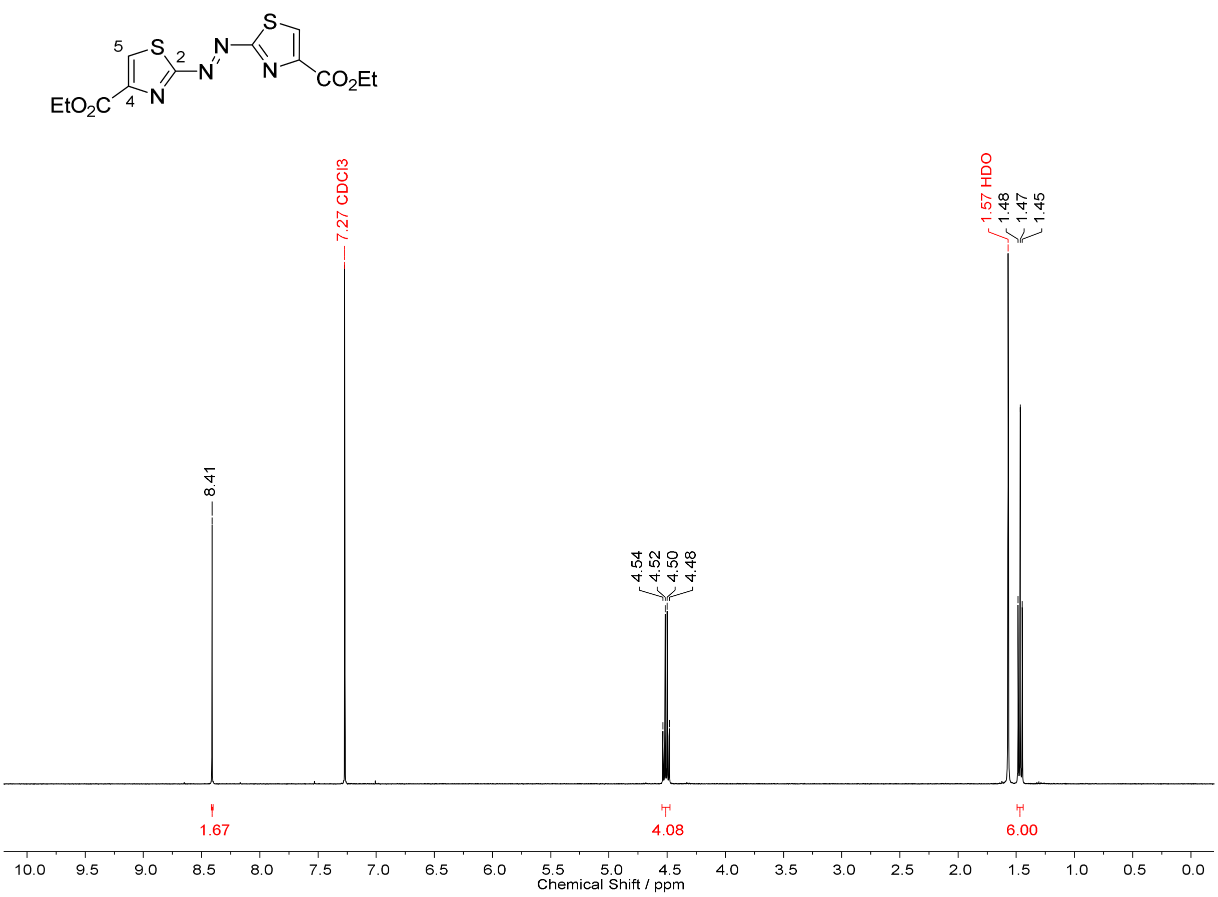

Figure S10. ^{1}H-NMR spectrum (400 MHz) of derivative **2g** in $CDCl_3$.

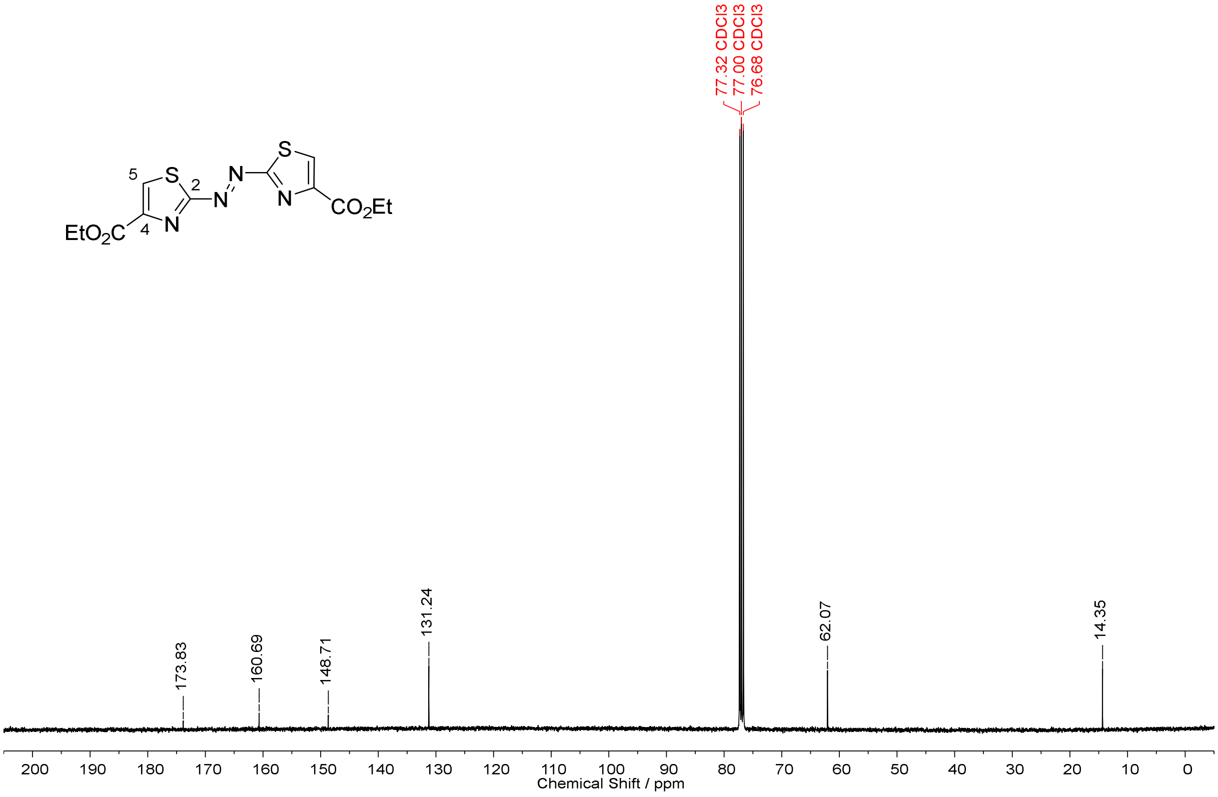

Figure S11. ^{13}C-NMR spectrum (100 MHz) of derivative **2g** in $CDCl_3$.

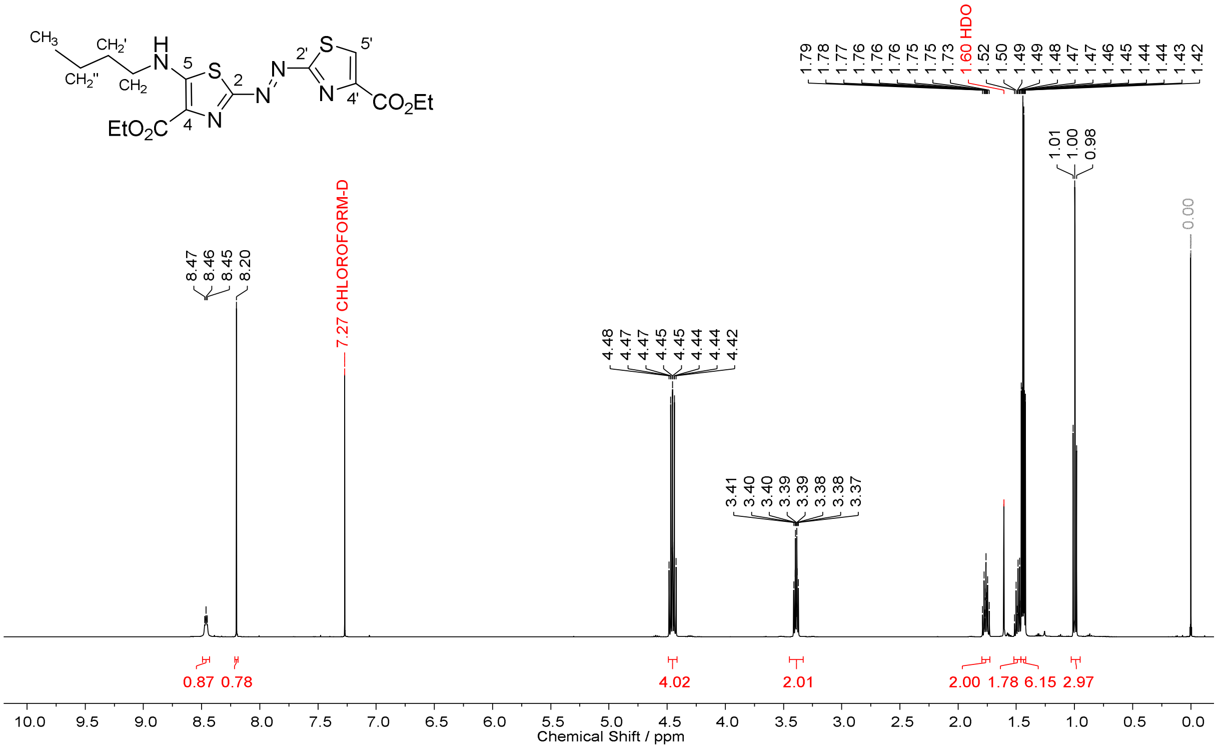

Figure S12. ^{1}H-NMR spectrum (500 MHz) of derivative **7a** in $CDCl_3$.

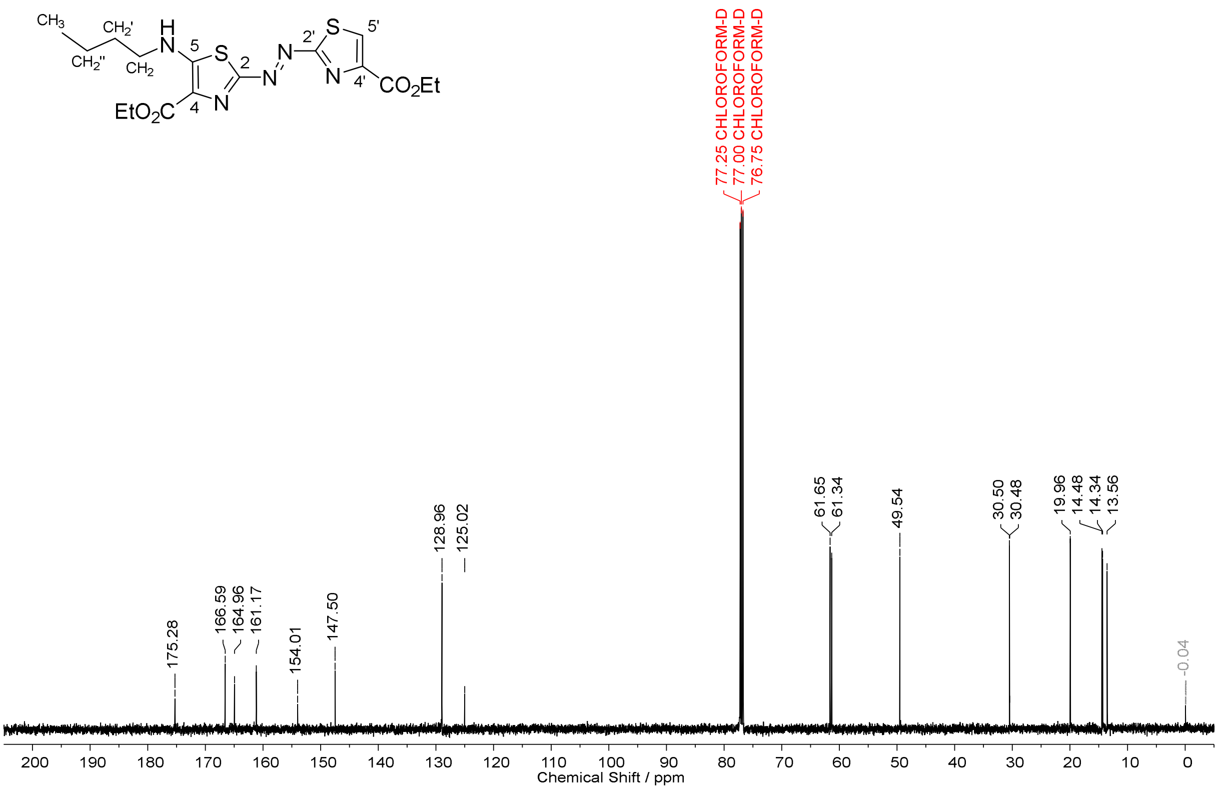

Figure S13. ^{13}C-NMR spectrum (125 MHz) of derivative **7a** in $CDCl_3$.

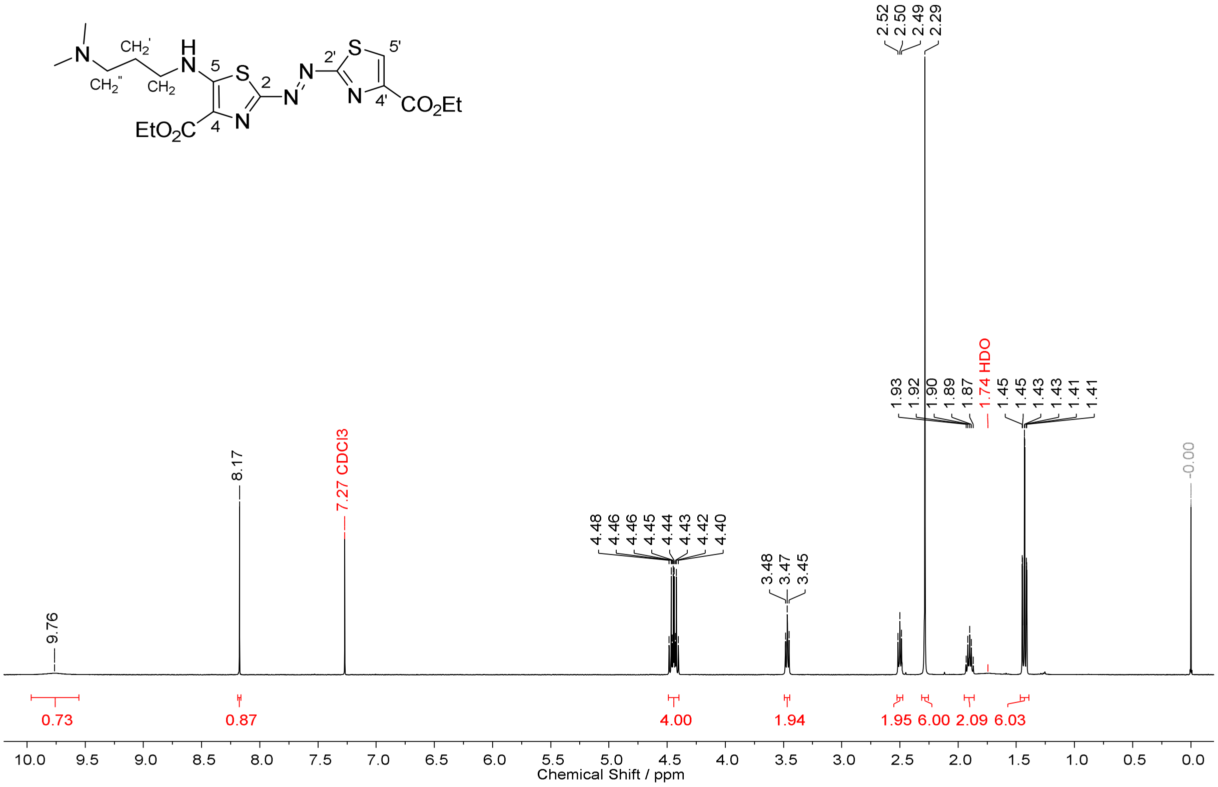

Figure S14. ^{1}H-NMR spectrum (400 MHz) of derivative **7b** in $CDCl_3$.

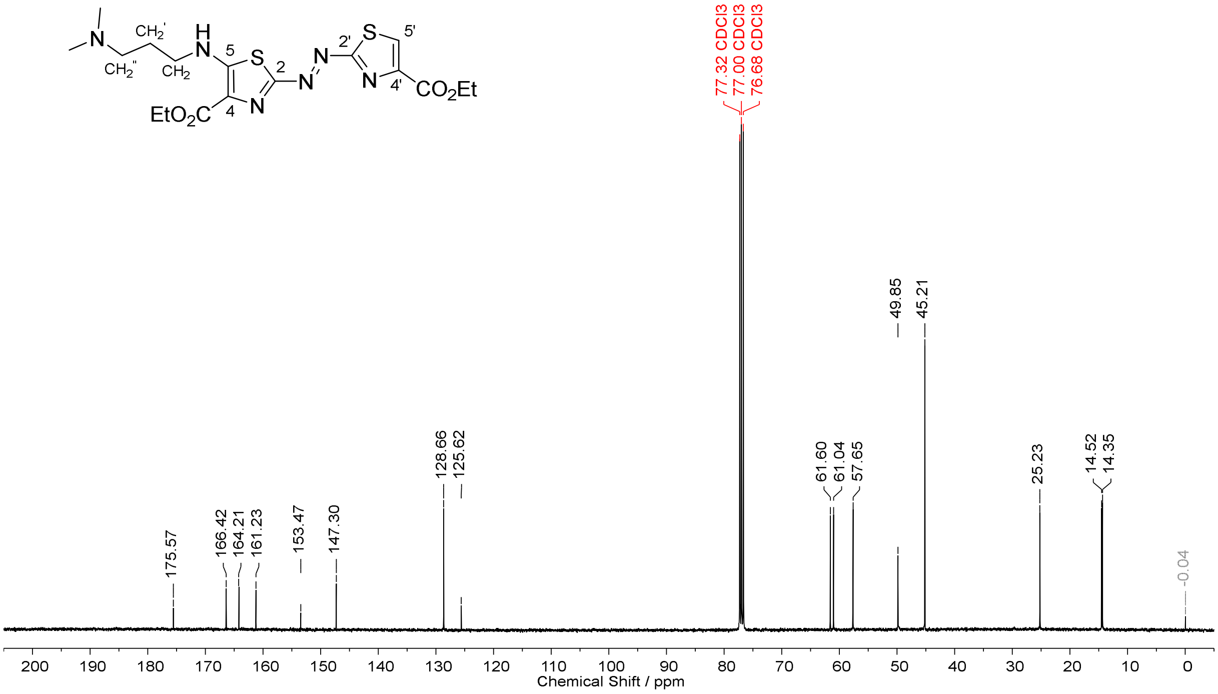

Figure S15. ^{13}C-NMR spectrum (100 MHz) of derivative **7b** in $CDCl_3$.

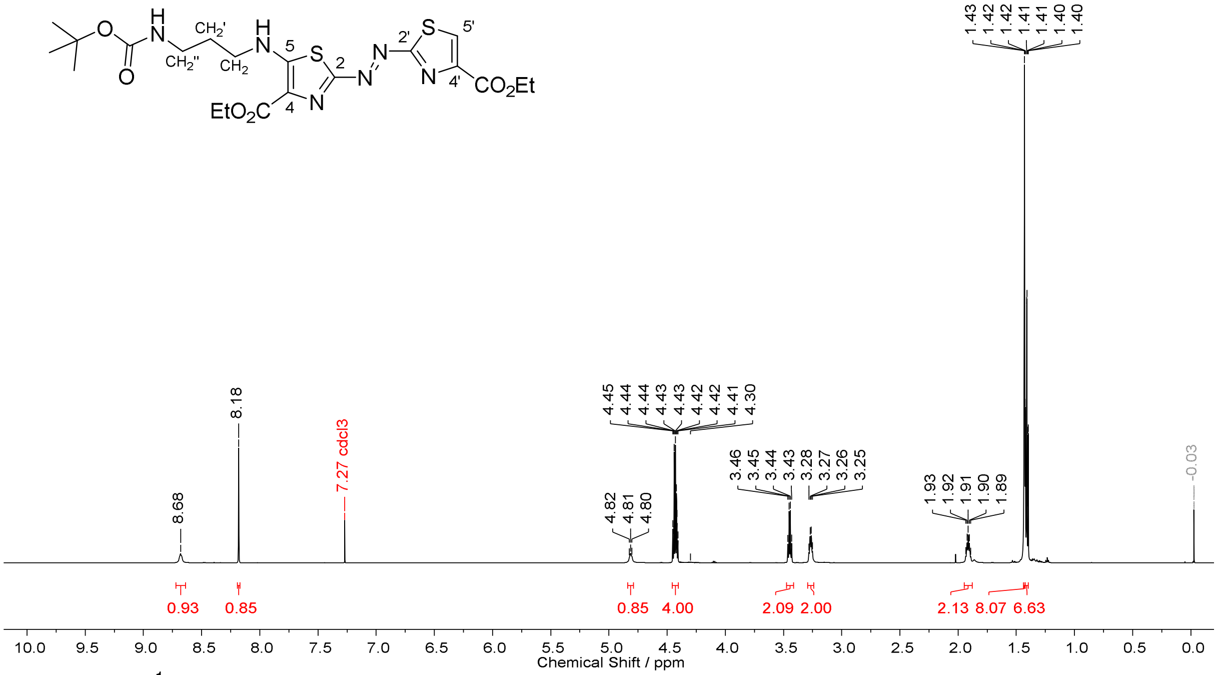

Figure S16. ^{1}H-NMR spectrum (600 MHz) of derivative **7c** in $CDCl_3$.

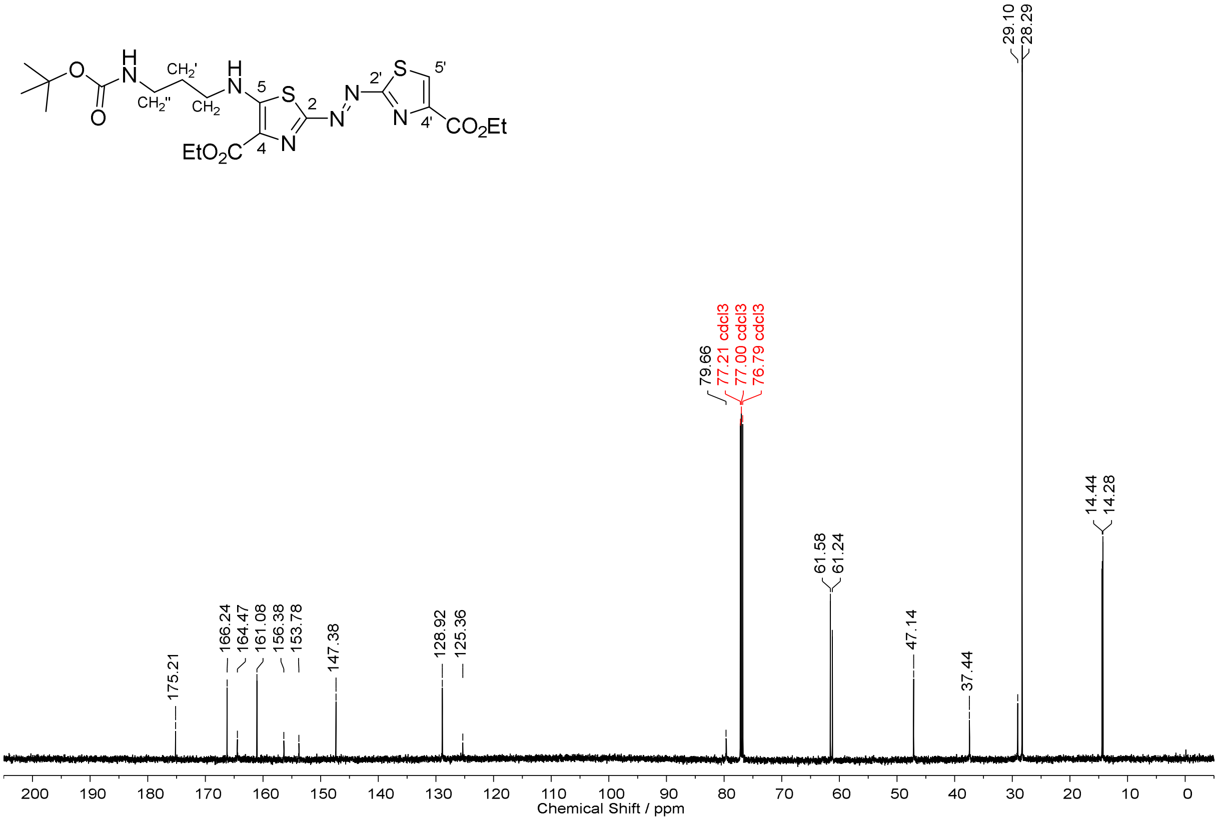

Figure S17. ^{13}C-NMR spectrum (150 MHz) of derivative **7c** in $CDCl_3$.

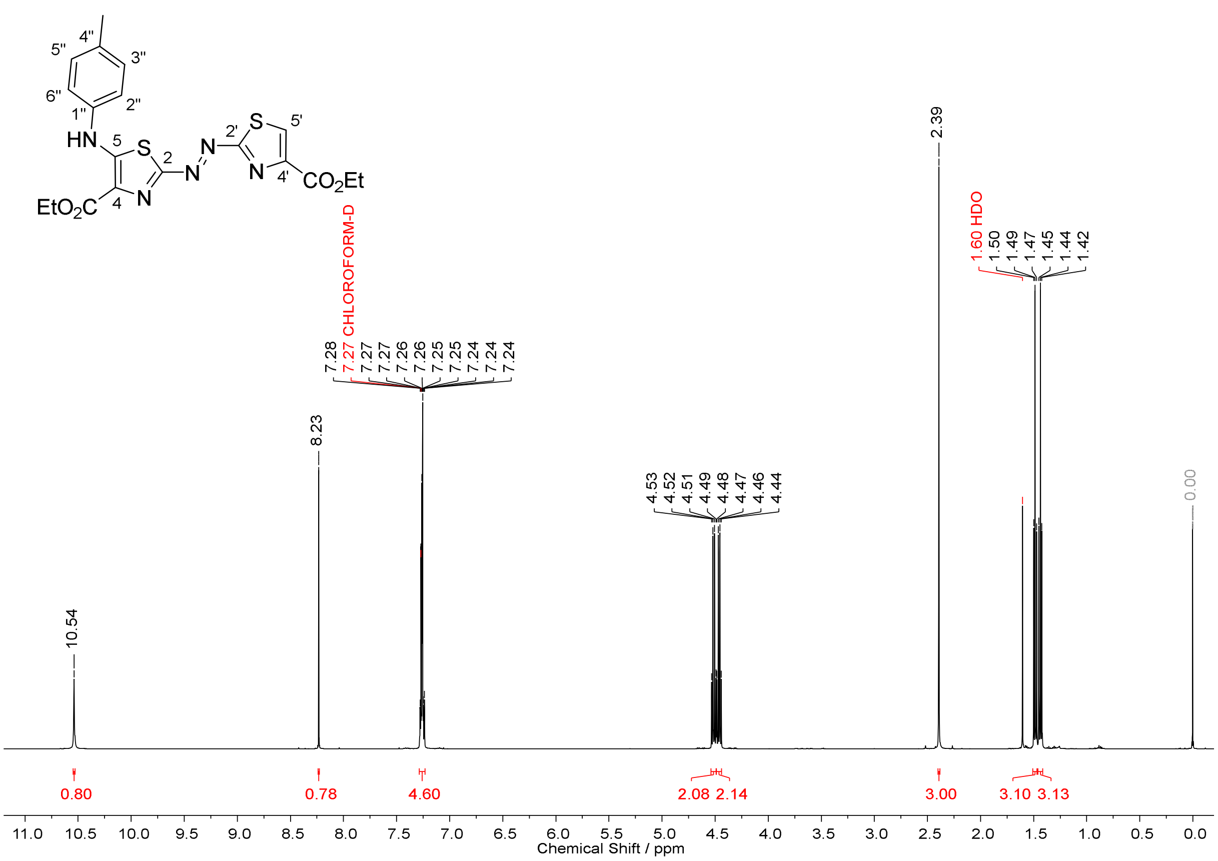

Figure S18. ^{1}H-NMR spectrum (500 MHz) of derivative **7d** in $CDCl_3$.

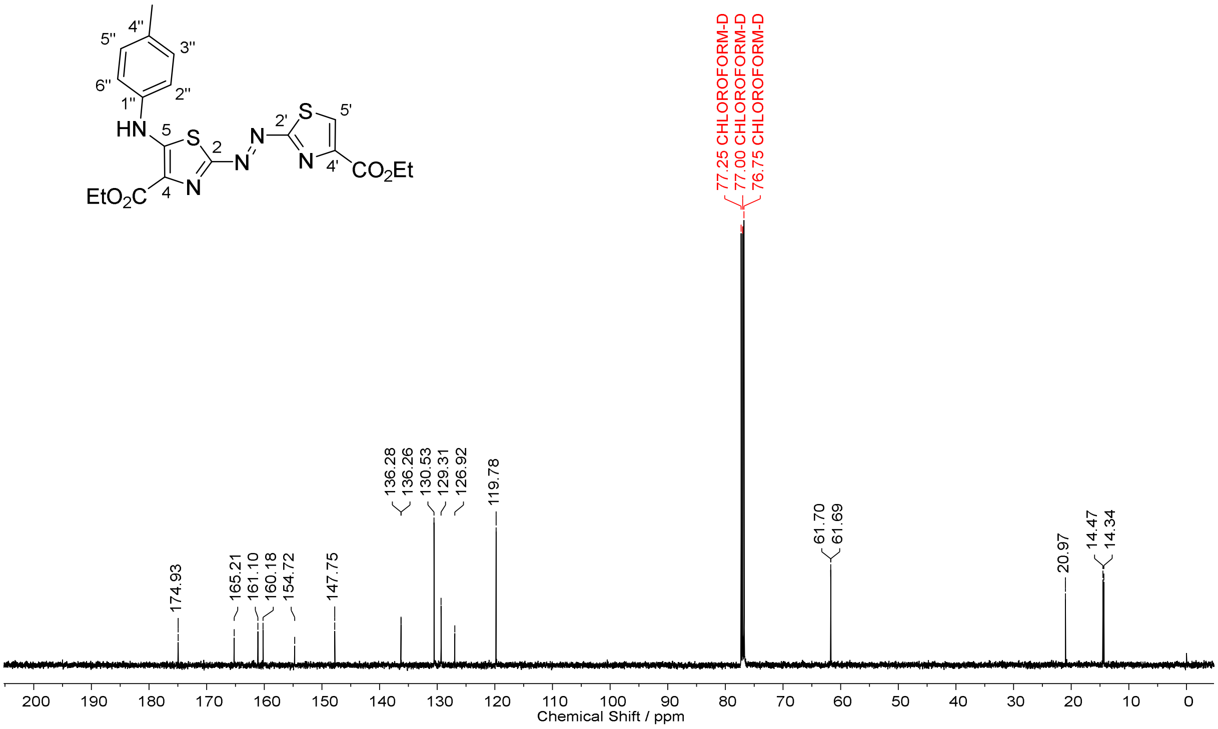

Figure S19. ^{13}C-NMR spectrum (125 MHz) of derivative **7d** in $CDCl_3$.

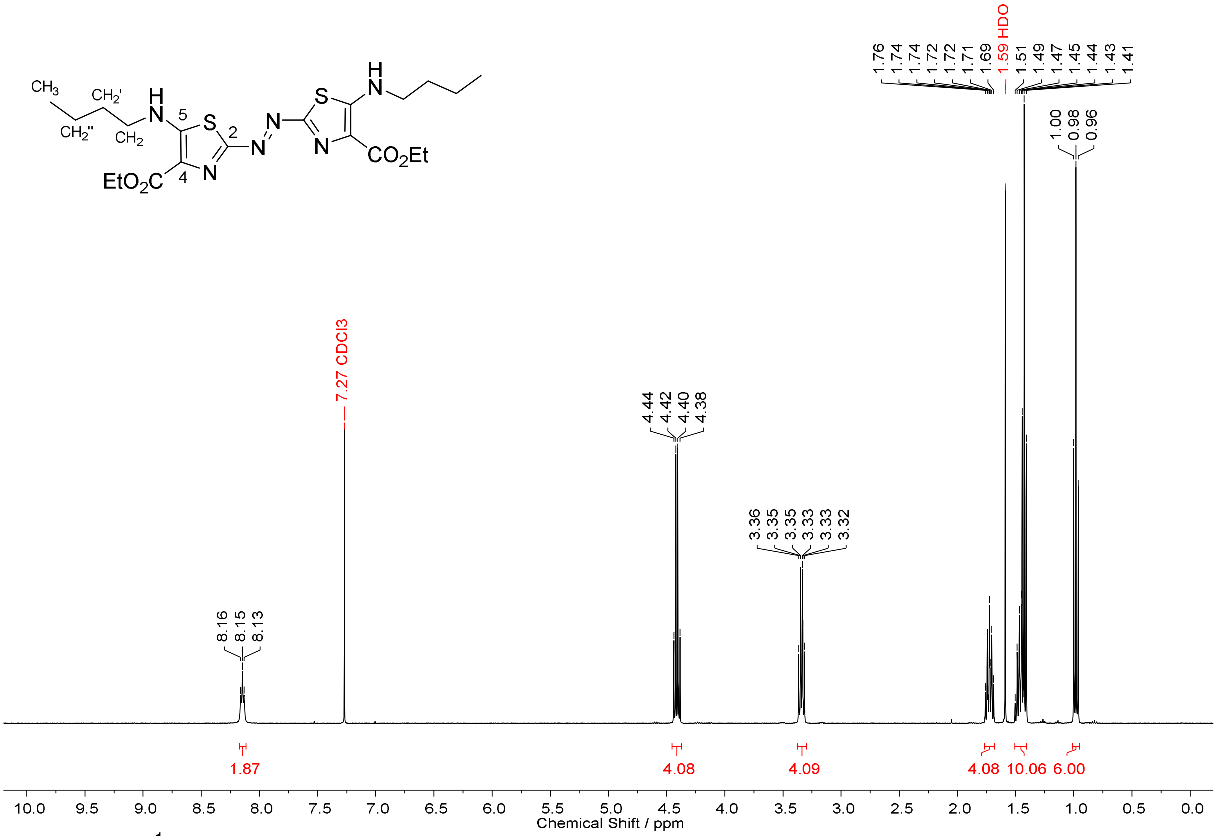

Figure S20. ^{1}H-NMR spectrum (400 MHz) of derivative **8a** in $CDCl_3$.

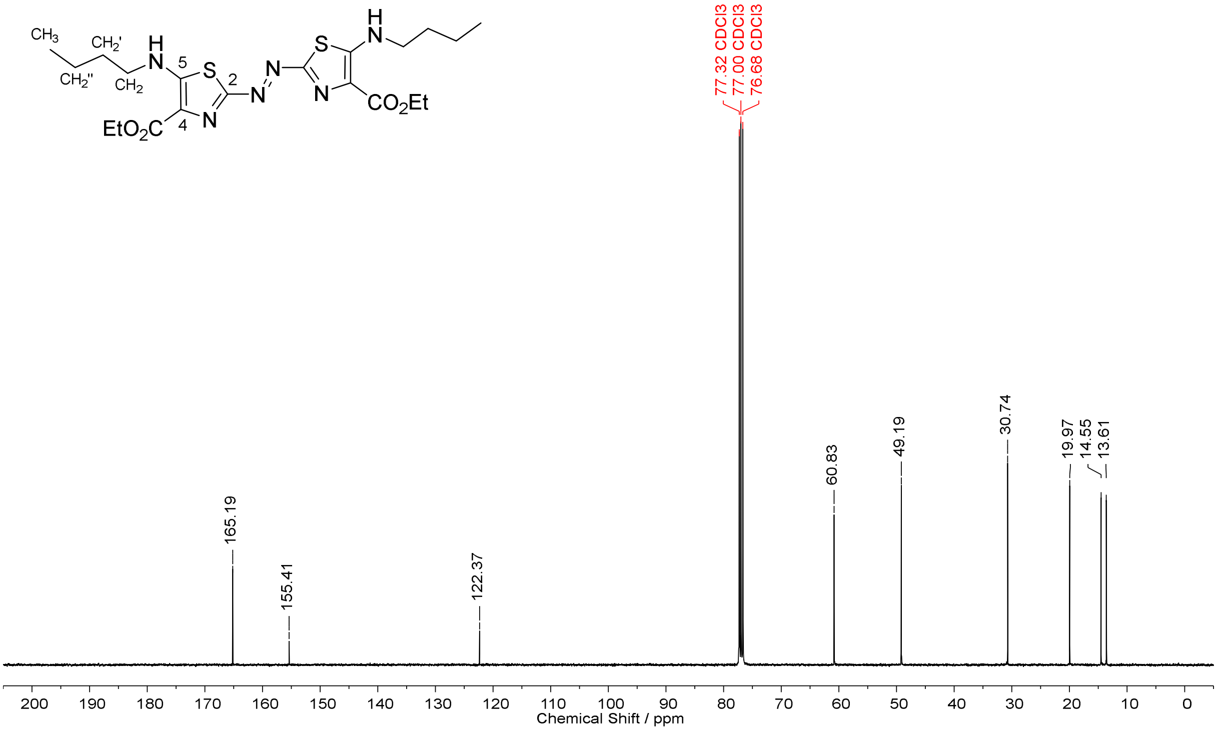

Figure S21. ^{13}C-NMR spectrum (100 MHz) of derivative **8a** in $CDCl_3$.

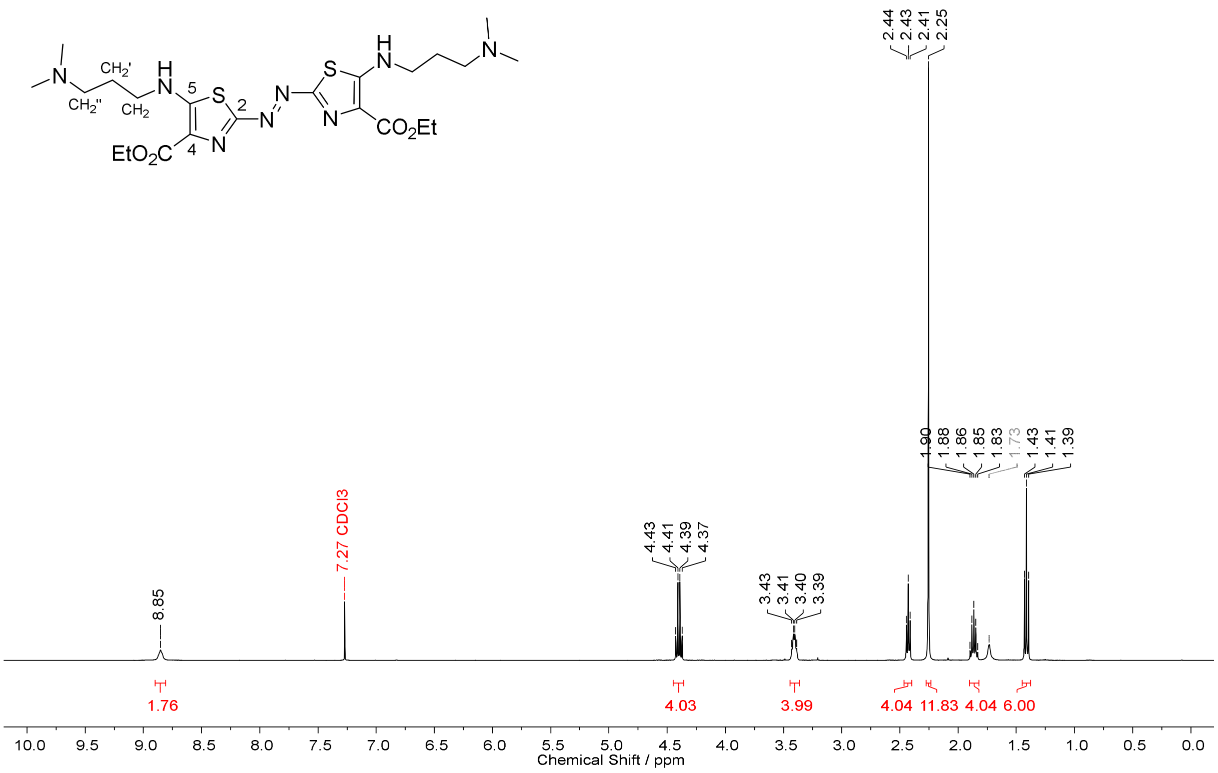

Figure S22. ^{1}H-NMR spectrum (400 MHz) of derivative **8b** in $CDCl_3$.

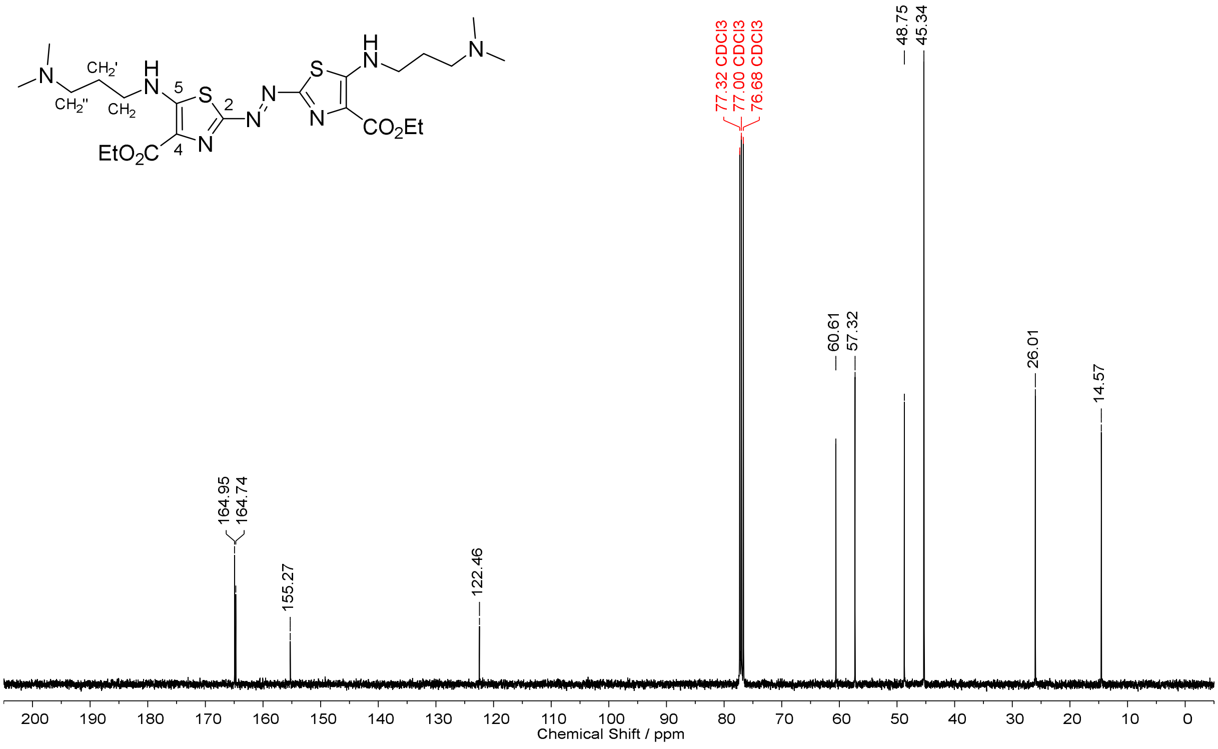

Figure S23. ^{13}C-NMR spectrum (100 MHz) of derivative **8b** in $CDCl_3$.

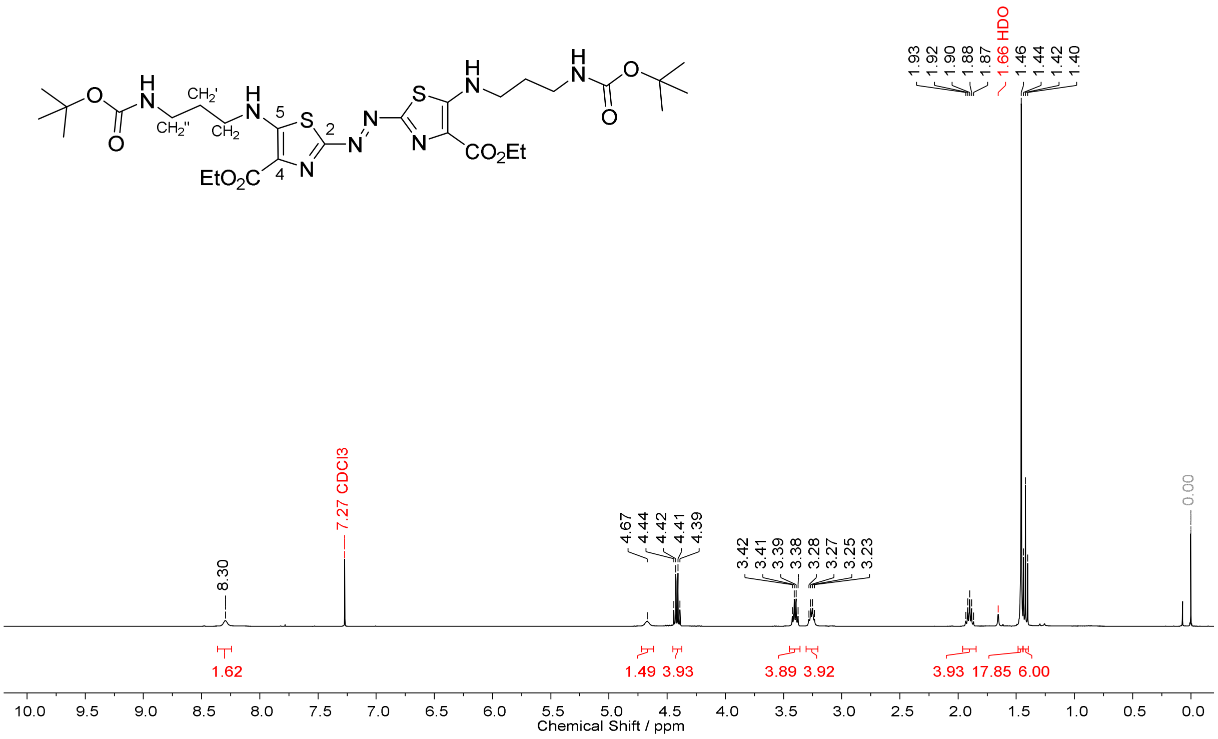

Figure S24. ^{1}H-NMR spectrum (400 MHz) of derivative **8c** in $CDCl_3$.

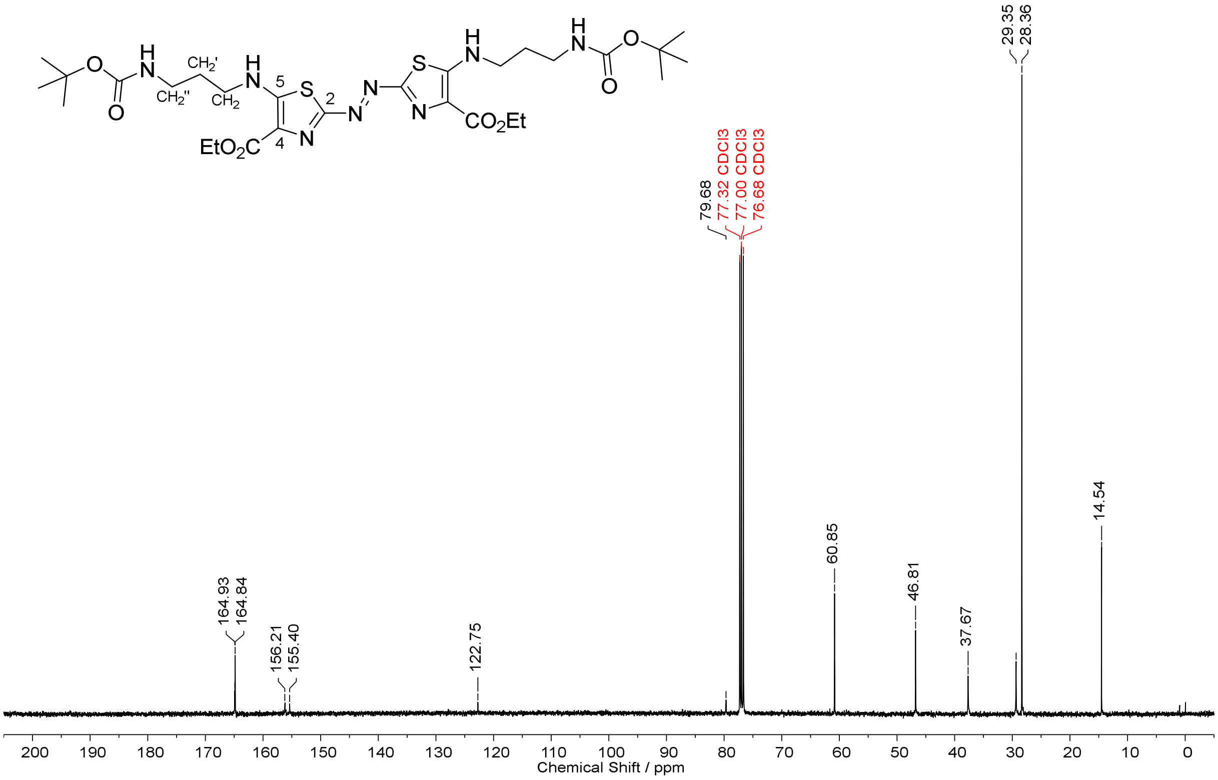

Figure S25. ^{13}C-NMR spectrum (100 MHz) of derivative **8c** in $CDCl_3$.

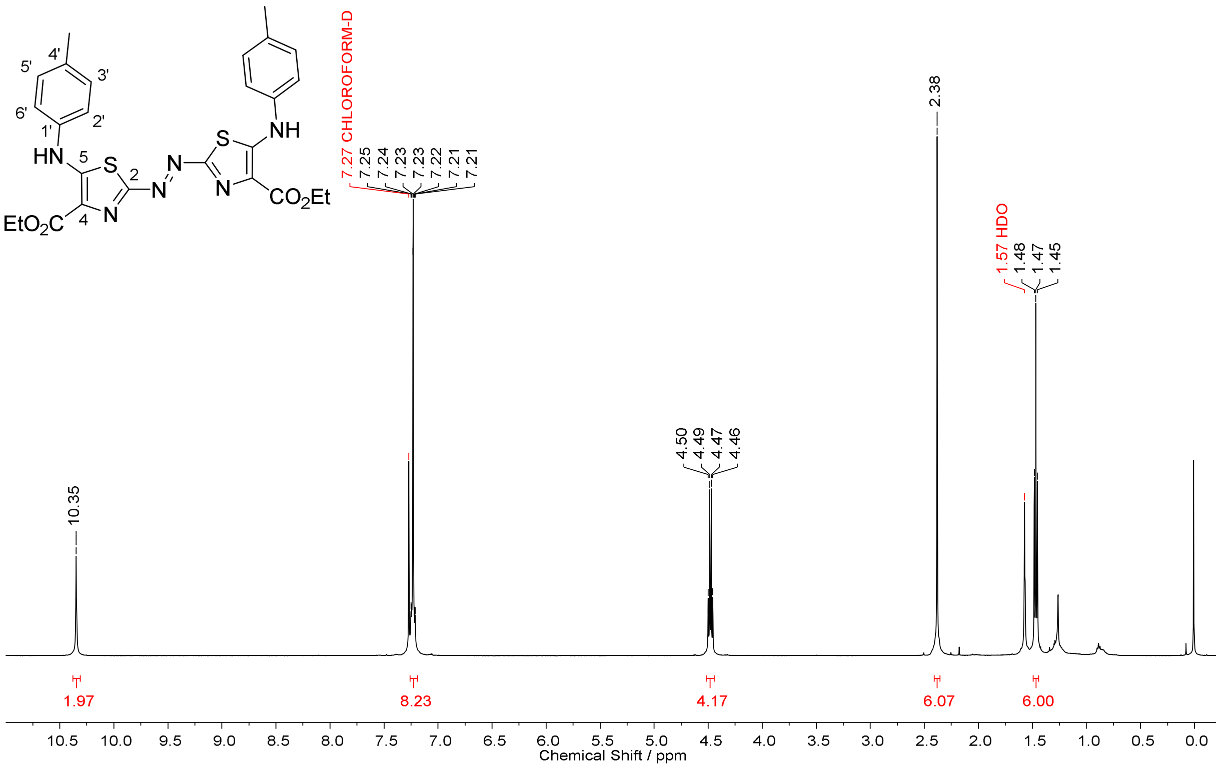

Figure S26. ^{1}H-NMR spectrum (500 MHz) of derivative **8d** in $CDCl_3$.

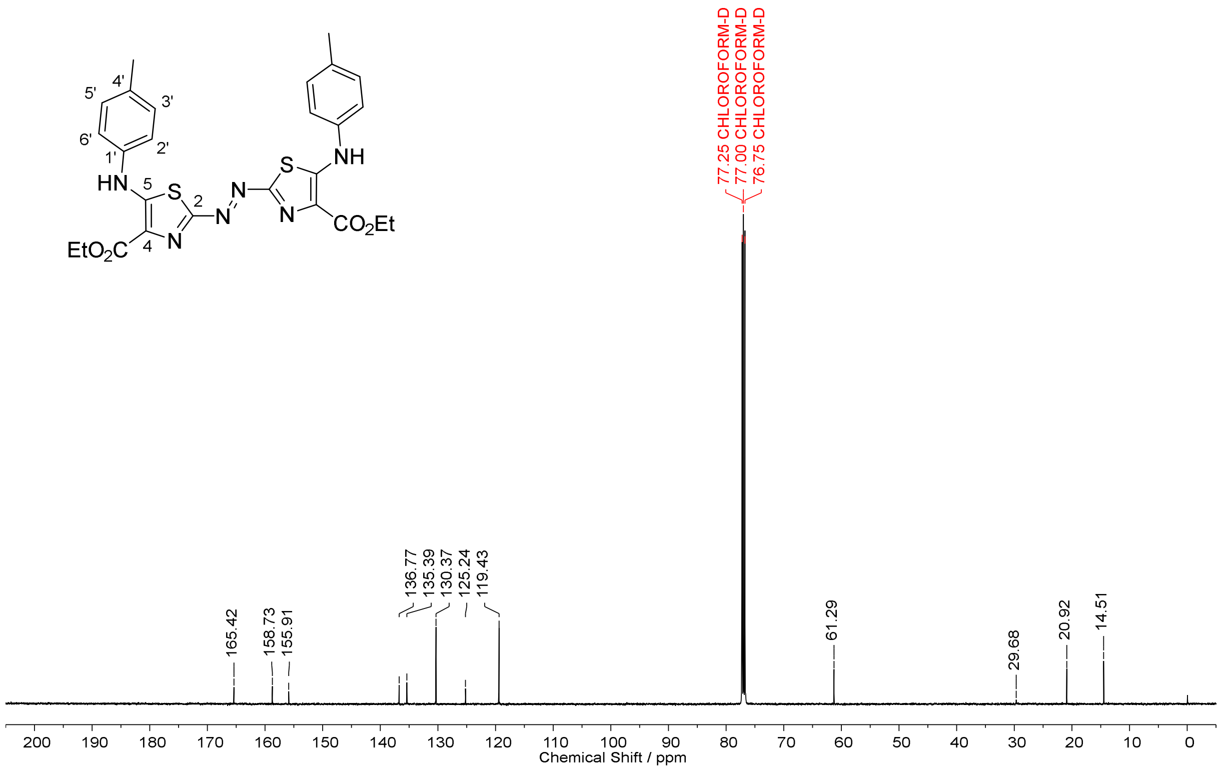

Figure S27. ^{13}C-NMR spectrum (125 MHz) of derivative **8d** in $CDCl_3$.

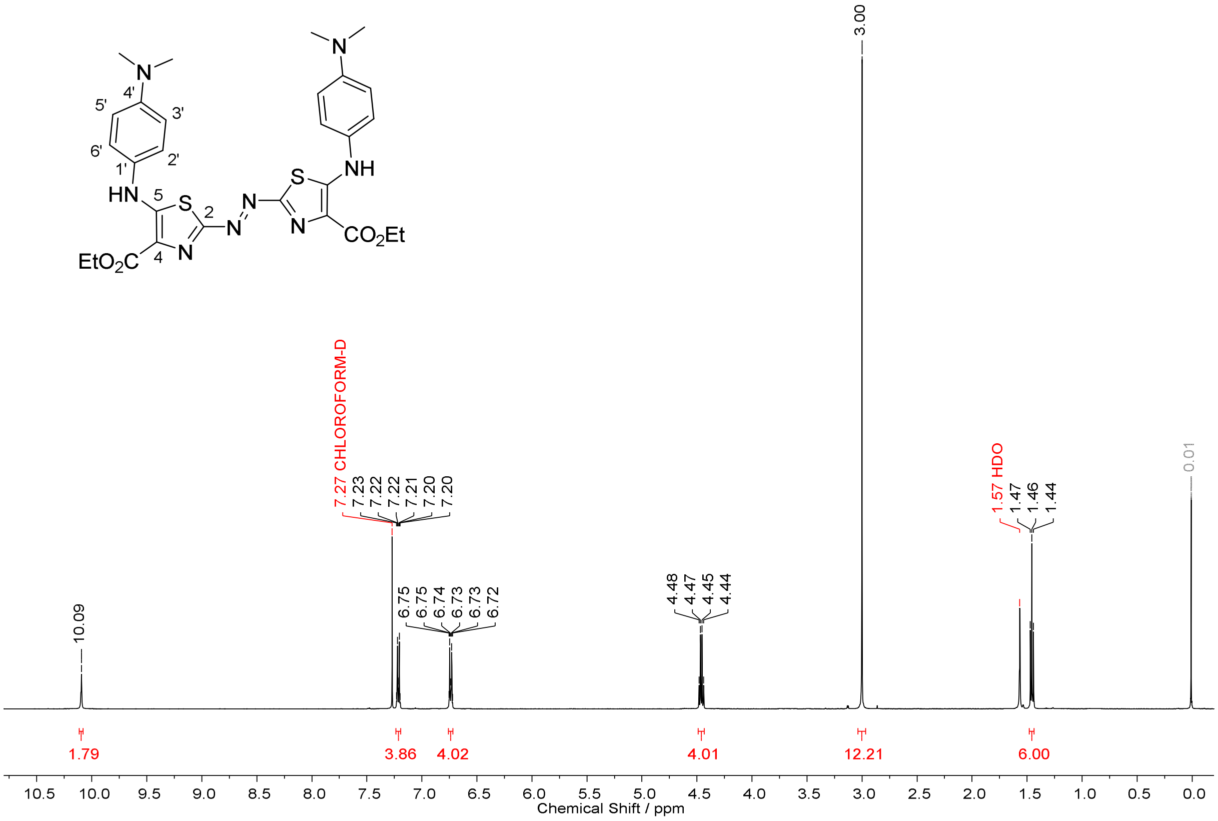

Figure S28. ^{1}H-NMR spectrum (500 MHz) of derivative **8e** in $CDCl_3$.

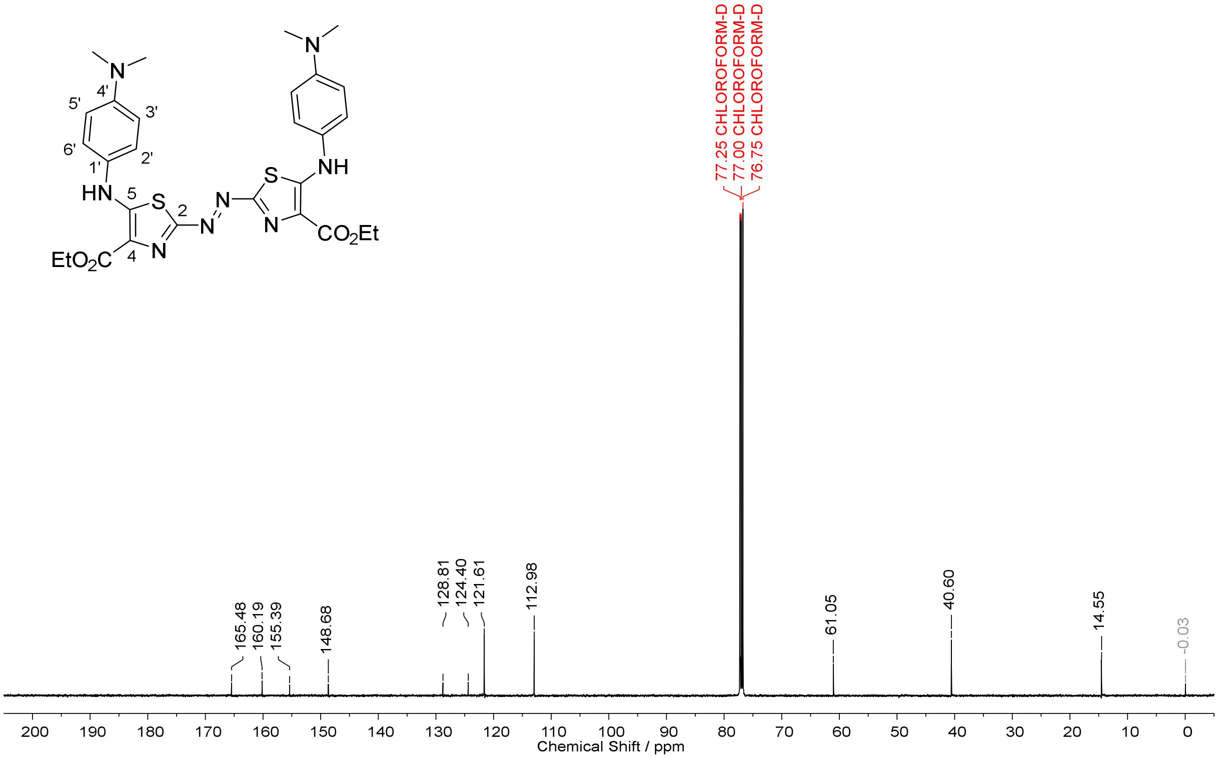

Figure S29. ^{13}C-NMR spectrum (125 MHz) of derivative **8e** in $CDCl_3$.

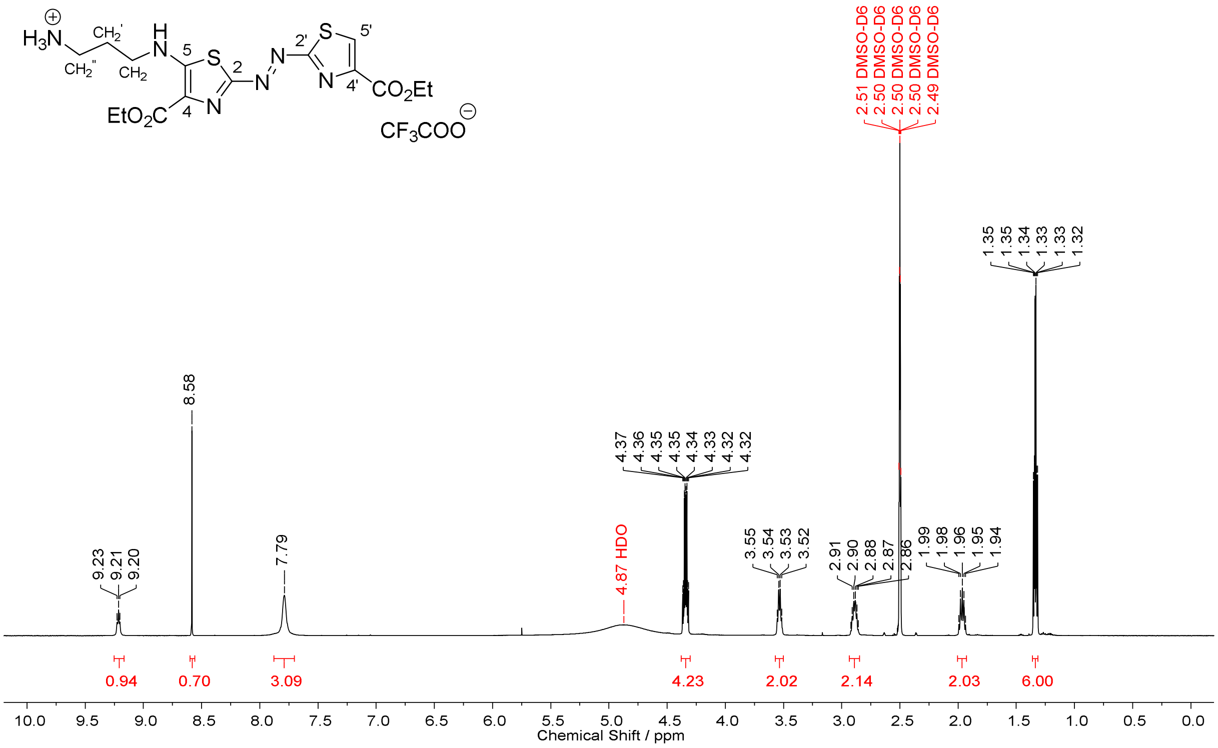

Figure S30. ^{1}H-NMR spectrum (500 MHz) of derivative **7f** in DMSO-d_6.

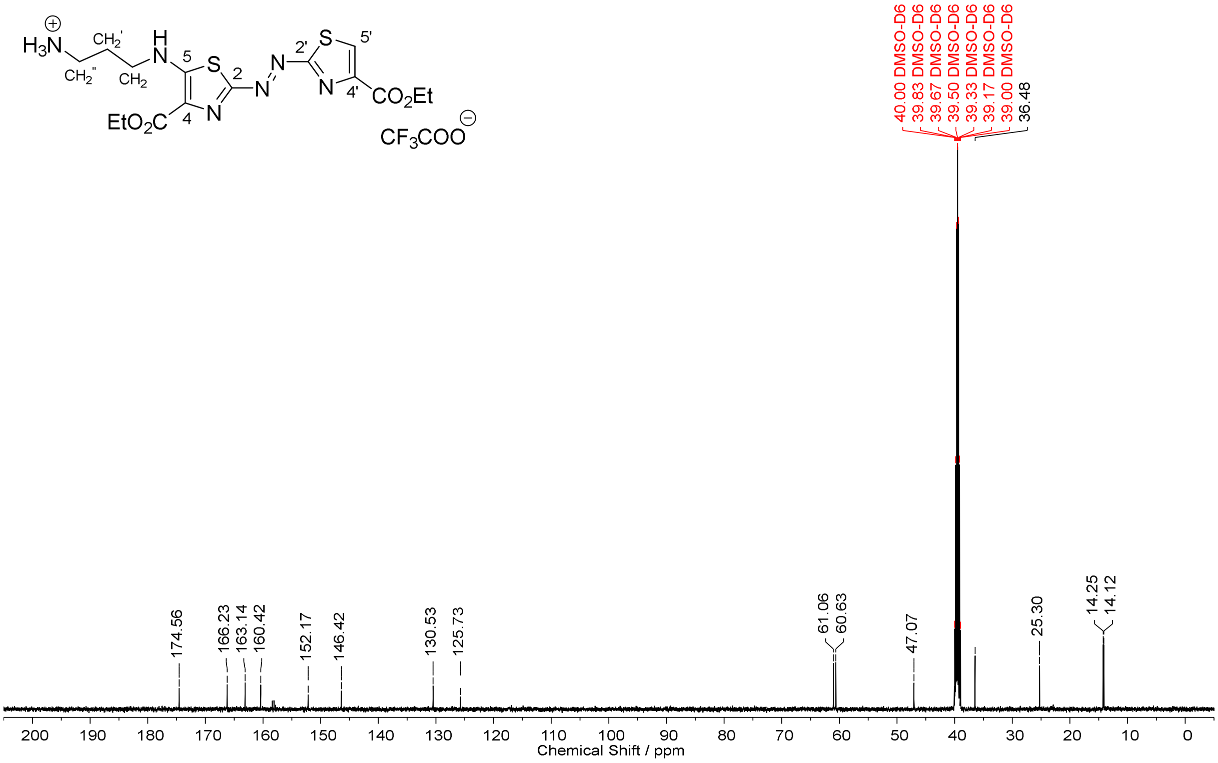

Figure S31. ^{13}C-NMR spectrum (125 MHz) of derivative **7f** in DMSO-d_6.

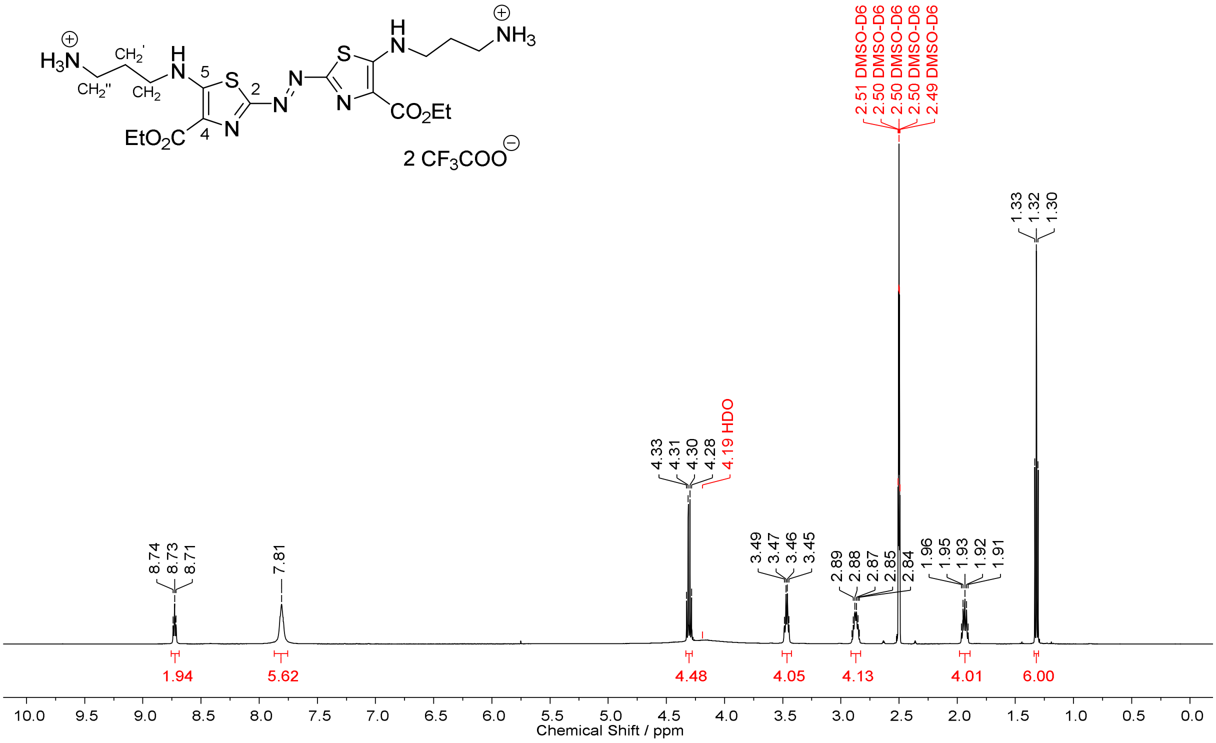

Figure S32. ^{1}H-NMR spectrum (500 MHz) of derivative **8f** in DMSO-d_6.

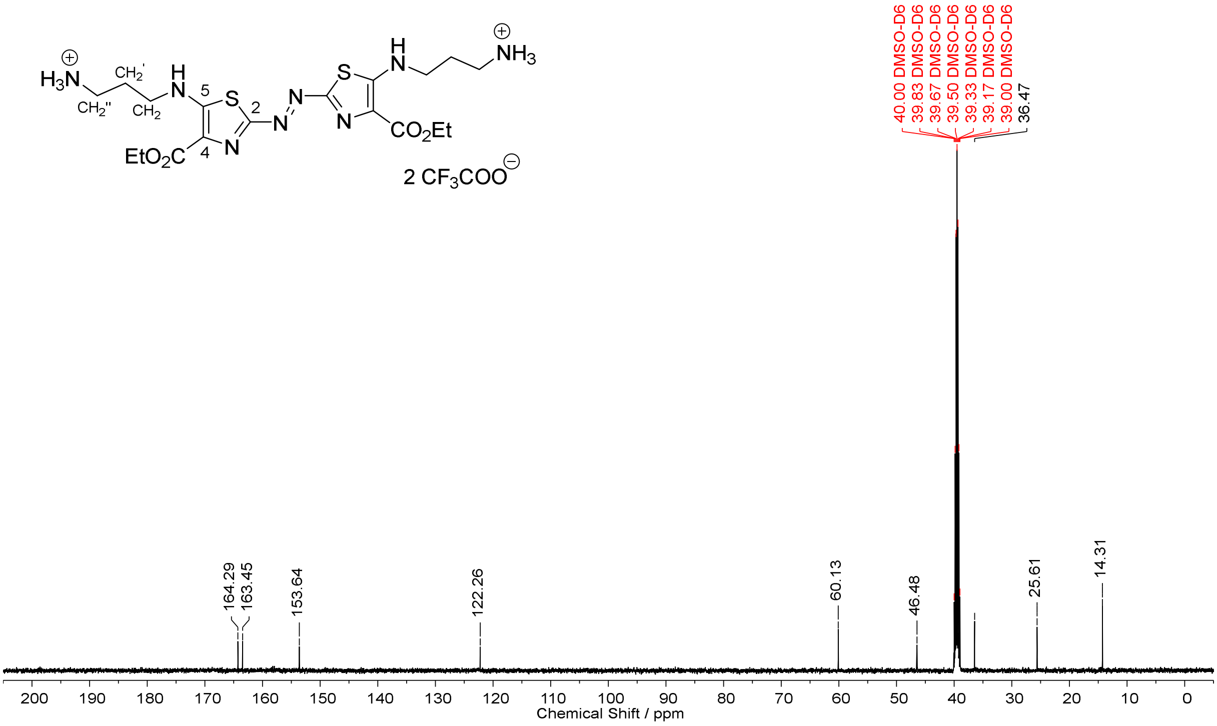

Figure S33. ^{13}C-NMR spectrum (125 MHz) of derivative **8f** in DMSO-d_6.

4 References

[1] D. Magde, J. H. Brannon, T. L. Cremers, J. Olmsted, *J. Phys. Chem.* **1979**, *83*, 696–699.

[2] a) G. A. Crosby, J. N. Demas, *J. Phys. Chem.* **1971**, *75*, 991–1024; b) B. Valeur, M. N. Berberan-Santos, *Molecular Fluorescence. Principles and Applications*, Wiley-VCH, Weinheim, **2012**.

[3] H. Beyer, A. Kreutzberger, *Chem. Ber.* **1951**, *84*, 482–485.

[4] D. Muller, I. Zeltser, G. Bitan, C. Gilon, *J. Org. Chem.* **1997**, *62*, 411–416.

[5] J. Catalán, *J. Phys. Chem. B* **2009**, *113*, 5951–5960.
